# Integrating emergency risk communication (ERC) into the public health system response: Systematic review of literature to aid formulation of the 2017 WHO Guideline for ERC policy and practice

**DOI:** 10.1371/journal.pone.0205555

**Published:** 2018-10-31

**Authors:** Ayan Jha, Leesa Lin, Sarah Massin Short, Giorgia Argentini, Gaya Gamhewage, Elena Savoia

**Affiliations:** 1 Emergency Preparedness, Research, Evaluation & Practice (EPREP) program, Division of Policy Translation & Leadership Development, Harvard T. H. Chan School of Public Health, Boston, MA, United States of America; 2 Interventions and Guidance, Infectious Hazard Management, Health Emergencies Programme, World Health Organization, Genève, Switzerland; 3 Department of Biostatistics, Harvard T. H. Chan School of Public Health, Boston, MA, United States of America; University of Memphis, UNITED STATES

## Abstract

The World Health Organization (WHO) commissioned a systematic review of literature to facilitate evidence syntheses for the development of emergency risk communication (ERC) guidelines for its member states. The goal of this review was to integrate ERC best practices into governmental and non-governmental health systems for all emergencies of public health concern, by addressing three questions: (1) to identify best practices for the integration of ERC into national and international public health preparedness; (2) to identify mechanisms to establish effective intra-agency, inter-agency, and/or cross-jurisdictional information sharing; and (3) to identify methods to coordinate risk communication activities between responding agencies across organizations and levels of response. The review covered scientific and grey literature publications between January 2003 and February 2016, and searches were conducted in 17 English language electronic libraries besides Chinese, Portuguese and Spanish language databases. A mixed deductive-inductive process was used to synthesize findings across studies through identifying thematic areas. While 8,215 articles were initially retrieved, after a sequential screening process, the final evidence syntheses comprised of 21 articles for question (1) and 24 for questions (2) and (3) combined (due to overlap of themes). The *confidence in findings* was assessed by the Qualitative Evidence Syntheses (GRADE-CERQual) tool. PRISMA guidelines were followed to the extent possible given the limitations inherent to a review largely based on qualitative studies. The identified literature was very context-specific and referred to mechanisms, practices from the field, and recommendations that were derived from planning or response efforts implemented at the national or local levels in specific countries. Integration of ERC functions into public health emergency preparedness, planning and response activities was influenced by reforming components of the leadership structure when needed, modifying organizational factors, and nullifying restrictions (including amending laws/ regulations) that might have been an obstacle to the timely release of information. Exercises and trainings were recognized as effective strategies to identify the barriers and successes in this process of integration. Key elements to enhance information sharing and coordination across organizations included the creation of networks, task-forces and committees across disciplines, organizations and geographic areas. Engagement of local stakeholders was also important to guarantee the flow of information up and down the incident command system. On the whole, few empirical studies, especially from low- and middle-income countries, related to the WHO research questions, demonstrating the need for research in these areas. To facilitate an accurate identification of the gaps, the authors suggest integrating current findings with case studies across the WHO regions to better understand the specific evidence that is needed in practice across the multitude of ERC functions.

## Introduction

Emergency risk communications (ERC) consist of "*the real-time exchange of information*, *advice and opinions*" between decision-makers, experts and the general public. [1] Over the last decade, nations have increasingly faced challenges in acquiring, processing and communicating information to protect the physical, social and economic wellbeing of their citizens during emergency situations. This challenge is in part due to the lack of evidence on how to best communicate among responding agencies and with the public. [2]

ERC is one of the eight core functions that World Health Organization (WHO) member states are required to fulfill as part of the International Health Regulations (IHR) [3]. All types of public health emergencies, ranging from infectious diseases outbreaks and pandemics to weather related events and manmade disasters, present national leaders with the challenge of communicating risk to the affected populations while maintaining trust, transparency and consistency of messages. While there are existing best practices and training in the field of ERC, there are few comprehensive, evidence-based, systems-focused principles and guidelines to support its practice. In response to this need, and recognizing that access to information is a fundamental right of an affected population, the WHO established a Guideline Development Group (GDG) in 2015 to steer the development of guidance on ERC for its member states. [4] This guideline has been recently published by the WHO, and provides advice and direction on how member states can integrate the best practices of risk communication into critical governmental and non-governmental health systems for all emergencies of public health concern (natural/man-made disasters, infectious disease epidemics/pandemics, and terrorism). [5]

The WHO commissioned the Emergency Preparedness, Research, Evaluation and Practice (EPREP) program at the Harvard T.H. Chan School of Public Health to conduct a systematic review of literature to facilitate evidence syntheses for the development of ERC guidelines for its member states (the full report has been made available by the WHO). [6] The goal of this review was to integrate ERC best practices into governmental and non-governmental health systems for all emergencies of public health concern, by addressing three questions: (1) to identify best practices for the integration of ERC into national and international public health preparedness; (2) to identify mechanisms to establish effective intra-agency, inter-agency, and/or cross-jurisdictional (such as cross-border; national with sub-national jurisdictions, etc.) information sharing; and (3) to identify methods to coordinate risk communication activities between responding agencies across organizations and levels of response. It was envisaged that answering these questions would facilitate the integration of knowledge regarding the effectiveness of ERC practices and structures into the WHO guideline development process.

Recognizing that the evidence answering these questions was more likely to be qualitative or mixed-methods research, the WHO developed the "Setting, Perspective, phenomenon of Interest, Comparison, Evaluation of impact" (SPICE) format to facilitate the interpretation of these questions and guide development of search terminology. The detailed breakdown of the SPICE format for each question is provided in the published WHO Guideline. [5]

## Methods

### Evidence acquisition

The review was primarily conducted for all scientific and grey literature in English, Chinese, Portuguese and Spanish language databases, between January 1, 2003 and February 7–9, 2016 (specific dates mentioned with the list of databases in Tables 1–3). Fourteen scientific and three grey literature English language electronic libraries were searched, and specific searches were carried out for publications in Chinese ("China Academic Journals Full-text" and "China National Knowledge Infrastructure" databases), Portuguese ("LILACS/SCielo" and "Mirage—Fiocruz" databases) and Spanish ("REDYLAC/SCielo" database). Manual cross-referencing of articles judged to be relevant to the questions of interest was also conducted for additional publications.

**Table 1:** Detailed search strategies for English language scientific databases.

	Source	Search Strategy
1.	Medline (PubMed) February 7, 2016	("Disaster Planning"[mesh] OR "Civil Defense"[majr] OR "Disasters"[mesh] OR "Disease Outbreaks"[Mesh] OR pandemic*[tiab] OR epidemic*[tiab] OR outbreak*[tiab] OR disaster*[tiab] OR emergency planning[tiab] OR emergency preparedness[tiab]) OR "preparedness"[tiab] OR "mitigation"[tiab] OR "influenza, human"[mesh] OR "ebola"[tiab] OR "terrorism"[mesh] OR "floods"[tiab] OR "earthquake"[tiab] OR "hurricane"[tiab] OR "cyclone"[tiab] AND ("Communication"[Mesh] OR communication*[tiab]) AND ("Risk"[mesh] OR "Risk Reduction Behavior"[mesh] OR "Risk Management"[mesh] OR risk[tiab]) AND ("2003/01/01"[PDAT]: "3000/12/31"[PDAT])
2.	Cochrane Databases February 7, 2016	Cochrane Central Register of Controlled Trials: Issue 1 of 12, January 2016 *Publication Year from 2003 to 2016*, *in Cochrane Reviews (Reviews and Protocol)*, *Other Reviews*, *Trials*, *Methods Studies*, *Technology Assessments*, *Cochrane Groups* (0) Title, Abstract, Keywords: Disaster AND communication Outbreak AND communication Epidemic AND Communication Pandemic AND Communication Preparedness AND communication Terrorism AND communication
3.	PDQ-Evidence February 7, 2016	("Disaster Planning" OR "Disease Outbreaks" OR pandemic OR epidemic OR outbreak OR disaster OR emergency planning OR emergency preparedness OR preparedness OR mitigation OR influenza OR Ebola OR terrorism OR flood OR earthquake OR hurricane OR cyclone) AND (communication) AND (Risk OR "Risk Reduction Behavior" OR "Risk Management") In: Title or Abstract Year: 2003–2016
4.	WHO Global Health Library February 8, 2016	("Disaster Planning" OR "Disease Outbreaks" OR pandemic OR epidemic OR outbreak OR disaster OR. emergency planning OR emergency preparedness OR preparedness OR mitigation OR influenza OR Ebola OR terrorism OR flood OR earthquake OR hurricane OR cyclone) AND (communication) AND (Risk OR "Risk Reduction Behavior" OR "Risk Management") In: Title, Abstract, Subject; EXCLUDE MEDLINE (1056 searches); 2003 -present
5.	Social Sciences Research Network (SSRN) February 8, 2016	Search by Title, Abstract, Abstract ID & Keywords; All dates; All SSRN Networks 1.Disaster >> communication (search within results for Disaster search) 2. Hazard >> communication 3. Pandemic >> communication 4. Epidemic >> communication 5. Outbreak >> communication 6. Preparedness >> communication 7. Terrorism >> communication
6.	Embase February 8, 2016	('disaster planning'/exp OR 'civil defense'/exp OR 'disaster'/exp OR 'epidemic'/exp OR 'pandemic'/exp OR outbreak*:ab,ti OR disaster*:ab,ti OR 'emergency planning': ab,ti OR epidemic*:ab,ti OR pandemic*:ab,ti OR 'emergency preparedness': ab,ti) AND ('interpersonal communication'/exp OR 'medical information'/exp OR communication*: ab, ti) AND ('risk'/exp OR risk: ab,ti) AND [embase]/lim AND [2003–2016]/py
7.	CINAHL February 8, 2016	MH ("Disasters+" OR "Civil Defense" OR "Disease Outbreaks") OR TI (outbreak* OR epidemic* OR pandemic* OR disaster* OR "emergency planning" OR "emergency preparedness") OR AB (outbreak* OR epidemic* OR pandemic* OR disaster* OR "emergency planning" OR "emergency preparedness") AND MH ("Communication") OR TI (communication) OR AB (communication) AND MH ("Risk Management") OR TI (risk) OR AB (risk) 2003–2016
8.	PsycINFO February 8, 2016	DE ("Emergency Preparedness" OR DE "Disasters" OR "Natural Disasters" OR "Epidemics" OR "Pandemics") OR TI (outbreak* OR epidemic* OR pandemic* OR disaster * OR "emergency planning" OR "emergency preparedness") OR AB (outbreak* OR epidemic* OR pandemic * OR disaster* OR "emergency planning" OR "emergency preparedness") AND DE ("Communication" OR "Electronic Communication" OR "Interpersonal Communication" OR "Communications Media" OR "Mass Media" OR "Multimedia" OR "Social Media" OR "Telecommunications Media") OR TI (communication) OR AB (communication) AND DE ("Risk Management") OR TI (risk) OR AB (risk) Dates: 2003–2016
9.	Communication Abstracts February 8, 2016	TX (outbreak* OR disaster* OR epidemic* OR pandemic* OR "emergency planning" OR "emergency preparedness") AND TX (risk)
10.	ERIC February 8, 2016	DE ("Natural Disasters" OR "Emergency Programs" OR "Civil Defense") OR TI (outbreak* OR epidemic* OR pandemic* OR disaster* OR "emergency planning" OR "emergency preparedness") OR AB (outbreak* OR epidemic* OR pandemic* OR disaster* OR "emergency planning" OR "emergency preparedness") AND DE ("Organizational Communication" OR "Communication (Thought Transfer)" OR "Communication Strategies" OR "Computer Mediated Communication") OR TI (communication) OR AB (communication) AND DE ("Risk" OR "Risk Management") OR TI (risk) OR AB (risk)
11.	Applied Social Sciences Index and Abstracts (ASSIA) February 8, 2016	SU.EXACT("Avalanches" OR "Cyclones" OR "Disasters" OR "Drought" OR "Earthquakes" OR "Ecological disasters" OR "Famine" OR "Firestorms" OR "Floods" OR "Hurricanes" OR "Natural disasters" OR "Tornadoes" OR "Volcanoes" OR "Disaster management") OR ti (outbreak* OR epidemic* OR pandemic* OR disaster* OR "emergency planning" OR "emergency preparedness") OR ab(outbreak* OR epidemic* OR pandemic* OR disaster* OR "emergency planning" OR "emergency preparedness") AND SU.EXACT("Communication" OR "Risk communication") OR ti(communication) OR ab(communication) AND SU.EXACT("Risk communication" OR "Risk management") OR ti(risk) OR ab(risk) Dates: 2003–2016
12.	Sociological Abstracts February 9, 2016	SU.EXACT("Disasters" OR "Natural Disasters" OR "Disaster Preparedness" OR "Epidemics") OR ti(outbreak* OR epidemic* OR pandemic* OR disaster* OR "emergency planning" OR "emergency preparedness") OR ab(outbreak* OR epidemic* OR pandemic* OR disaster* OR "emergency planning" OR "emergency preparedness") AND SU.EXACT("Organizational Communication" OR "Communication" OR "Computer Mediated Communication" OR "Publicity") OR ti(communication) OR ab(communication) AND SU.EXACT("Risk") OR ti(risk) OR ab(risk) Dates: 2003–2016
13.	Web of Science February 9, 2016	Indexes = SCI-EXPANDED, SSCI, A&HCI, CPCI-S, CPCI-SSH, BKCI-S, ESCI Timespan = 2003–2016 TS = ("outbreak*" OR "epidemic*" OR "pandemic*" OR "disaster*" OR "emergency planning" OR "emergency preparedness") AND TS = (communication) AND TS = (risk)
14.	Russian Academy of Sciences Bibliographies February 9, 2016	TX (outbreak* OR disaster* OR epidemic* OR pandemic* OR "emergency planning" OR "emergency preparedness") AND TX (communication*)

**Table 2:** Detailed search strategies for English language grey literature databases.

	Source	Search Strategy
1.	Bielefeld Academic Search Engine February 8, 2016	Title search; Year: 2003–2016; Books/articles/journals/reports/papers/lectures/theses/reviews/primary data Disaster AND communication Outbreak AND communication Epidemic & Communication Pandemic & Communication Preparedness AND communication Terrorism AND communication
2.	PAIS International February 8, 2016	SU.EXACT("Disasters" OR "Natural Disasters" OR "Disaster Preparedness" OR "Epidemics") OR ti(outbreak* OR epidemic* OR pandemic* OR disaster* OR "emergency planning" OR "emergency preparedness") OR ab(outbreak* OR epidemic* OR pandemic* OR disaster* OR "emergency planning" OR "emergency preparedness") AND SU.EXACT("Risk Communication" OR "Communication" OR "Computer Mediated Communication" OR "Publicity") OR ti(communication) OR ab(communication) AND SU.EXACT("Risk" OR "Risk Communication") OR ti(risk) OR ab(risk) Date: 2003–2016
3.	Policy File February 9, 2016	risk communication disaster* risk communication epidemic* risk communication outbreak* risk communication "emergency planning" risk communication "emergency preparedness" subject(crisis management) keyword "risk communication"

**Table 3:** Detailed search strategies for Chinese/Mandarin, Portuguese and Spanish language databases.

Source	Search Strategy
**Chinese/ Mandarin:** 1. China Academic Journals Full-text Database 2. China National Knowledge Infrastructure (CNKI) February 9, 2016	主题=(风险+危机+应急+重大灾害+埃博拉+甲型流感+SARS+非典+非典型肺炎+公共危机+风灾+巨灾+中东呼吸综合症+地震+流感+雪+水+旱+涝+洪+ 突发公共卫生事件+突发公共事件+风险传播+风险沟通+风险信息+危机传播+危机沟通+危机信息+应急传播+应急沟通)*(传播+沟通)并且 关键词=(突发公共卫生事件+突发公共事件+风险传播+风险沟通+风险信息+危机传播+危机沟通+危机信息+应急传播+应急沟通)-高校-血-医患-护理-金融-生态-药-信息安全-保险-浅谈-论述-概述-品牌-HIV-急诊-急救-个人信息-sex 并且 年=(2016+2015+2014+2013+2012+2011+2010+2009+2008+2007+2006+2005+2004+2003) (精确匹配),Subjects: 预防医学与卫生学,感染性疾病及传染病,急救医学,公安行政工作,交通管理,社会科学理论与方法,新闻与传媒,图书情报与数字图书馆,市场研究与信息,管理学,领导学与决策学;No sorting; Search mode: Single-database search
**Portuguese:** 1. LILACS/SCielo 2. Mirage—Fiocruz February 9, 2016	a) NATURAL DISASTERS AND RISK COMUNICATION (DESASTRES NATURAIS E COMUNICACAO) b) EPIDEMICS/PANDEMICS AND RISK COMUNICATION (EPIDEMIAS E COMUNICACO DO RISCO) c) MASS EVENTS AND COMUNICATION OF RISK (EVENTOS DE MASSA E COMUNICACAO DO RISCO) d) OLIMPICS AND FOOTBALL WORLD CHAMPIONSHIP AND RISK COMUNICATION
**Spanish:** REDYLAC/SCielo February 9, 2016	“Epidemias y pandemias y desastres naturales y grandes eventos y comunicación del riesgo desde el 2003 Liliacs y Scielo"

The Portuguese and Spanish search engines cited above did not allow for the use of a complex search string, such as the one used in Medline. Therefore, multiple search strings had to be used, as described above

Since a key objective was to look for evidence in the low and middle-income countries, the search strategies in all global databases (like PubMed, Cochrane, WHO Library etc.) were expanded to also include publications in Arabic, French, Russian, Italian and Japanese, besides the above-mentioned languages (these additional languages were selected on the basis of linguistic expertise available to the researchers). The web pages of a number of governmental and non-governmental public health agencies from French, Spanish, and Portuguese speaking countries, as well as Italy (as the major Italian-speaking nation) were screened. The research team also communicated with several risk communication experts and academics across USA, Europe (England, Germany, Switzerland, members of European CDC), Asia (India, China, Japan, Bangladesh, Vietnam), Latin America (Portugal, Argentina) and the Middle East (Qatar) to substantiate the understanding of systemic gaps and political-cultural sensibilities in ERC, as well as to request guidance to find region-specific publications related to the WHO questions.

The articles retrieved from the initial search (n = 8,215) were independently screened by AJ and SS for English language articles (including English language abstracts of articles in Japanese and German), LL for Chinese articles, ES for Italian language articles, and GA and ES for Spanish and Portuguese language articles. No articles published in Arabic, French or Russian languages were identified, though it was possible that the English language abstract of such an article was included among those retrieved from the global databases. The detailed search strategies for English, Chinese, Portuguese and Spanish language databases are provided in Tables 1–3.

In the title review phase, the authors specifically screened for articles including the terms "communication" or related words (risk/ crisis communication) along with an event of interest like any natural or man-made disaster, any infectious disease outbreak or epidemic or pandemic, or any event of terrorism (or simulation studies on these scenarios). Articles were also included if the terms "risk management,” "preparedness," "preparedness exercise," or “knowledge” were mentioned in the title. In the next phase, the researchers evaluated 1899 abstracts to include only: (i) research based on an empirical study [results/recommendations based on some observation or experiment]; and (ii) research directly or indirectly providing information to address at least one of the three WHO questions. This screening ensured that only articles which clearly violated the above criteria were excluded. Subsequently, 880 full-text articles were reviewed to determine eligibility for inclusion based on the assessment that they addressed components of the WHO SPICE breakdown, the details of which can be found in the published WHO Guidelines (Annex 3: SPICE questions #1, 2 and 6). [5]

To determine if the research was addressing the WHO questions, two reviewers independently read the article (with the exception of articles in Chinese language), and based on the SPICE breakdown identified sentences (in the article) supported by empirical data that would provide information which directly answered a WHO question or was related to the question (indirectly answered the question). Subsequently the two reviewers met to discuss their judgment, and in case of disagreement a third reviewer was consulted. Furthermore all articles deemed to be indirectly related were discussed with the WHO team to make sure the information was related to the questions developed by WHO.

#### Categorization of articles

Study title, first author, year of publication, type of publication (i.e. journal article, organizational report etc.), country/ region of the study, study design (i.e. qualitative, quantitative, mixed-methods or case study, with subtypes as applicable), population/sample studied or addressed (i.e. general population, affected/ vulnerable groups, stakeholders etc …), the type of disaster/crisis situation described and the phase of disaster (i.e. prevention, preparedness, response, recovery/rehabilitation) addressed were listed. The objectives of each study were noted, and the key topic area and principal findings and/or recommendations were carefully described. The relevance of an article to one or more of the WHO questions under consideration was critically judged and differences in opinion were resolved through consultations (as described earlier).

An article was defined to be "directly relevant" to a specific WHO question if: (1) it had an empirical study design, and (2) the findings and/or recommendations directly answered that particular WHO question. If an article did not satisfy both these criteria, it was coded as "indirectly relevant" and not included in the final evidence synthesis.

### Methods of evidence synthesis

Four methodological streams were identified to classify and characterize the selected articles: 1) quantitative (randomized group comparison, non-randomized group comparison and descriptive surveys), 2) qualitative (interviews, focus groups and textual/content analysis), 3) mixed-methods (use of both quantitative and qualitative techniques) and 4) case studies (description of the response to a particular emergency/crisis situation). The literature was then synthesized using the Best Fit Framework Synthesis described by Booth et al. [7] and Barnett-Page [8], which involves searching for existing theories/conceptual frameworks and creating a composite framework describing findings within each theme/concept. A mixed deductive-inductive process was used to synthesize findings across studies, starting with the SPICE components for each WHO question to develop the themes, and subsequently adding more thematic areas based on the literature.

For question 1, the syntheses of evidence is presented separately for English (n = 6) and Chinese language (n = 15) publications because of the different types of studies included in the two languages and difficulties in merging concepts and themes between the two. However, integration of the themes in a unified synthesis among articles in the two aforementioned languages was possible for questions 2 and 3, and therefore the evidence syntheses for these two questions (all language publications) have been presented together.

### Quality assessment of the evidence

A number of tools were applied to assess the quality of each study based on the methodology and study design: the Critical Appraisal Skills Program (CASP) tools were used to assess cohort studies and qualitative studies (including content analysis) [9], the Mixed Methods Appraisal Tool (MMAT) for studies using mixed methods [10], the Cochrane risk of bias tool for randomized interventional studies [11], the AMSTAR evaluation for systematic reviews [12], and the critical appraisal checklist for studies including surveys (The BMJ, Table E) [13]. Subsequently, *confidence in findings* was assessed by the use of the Qualitative Evidence Syntheses (GRADE-CERQual) tool, and a level of confidence was assigned to each study (high/ moderate/ low/ very low) based on methodological limitations, relevance, coherence and adequacy of data [14]. The GRADE-CERQual assessment was completed both for individual articles (S1–S3 Tables) and for the syntheses of findings derived from multiple articles within each WHO question (Tables 4–6). GRADE principles were adapted for application to descriptive quantitative studies and GRADE CERQual principles were applied to mixed-method studies. Neither adaptation has been approved by the tool originators. Please refer to the WHO Guideline for ERC policy and practice 2017 [5] for details.

**Table 4:** Synthesis of findings across methodological streams–Question 1, English language literature.

Thematic area of practice	Synthesis of findings	Citations	CERQual assessment of confidence in evidence
**Placement of ERC Functions in National Leadership Structure**	Renovations of existing organizational structures or creation of new ones are sometimes needed to achieve a better placement of ERC functions in the national leadership structure. As reported by Yen et al (2009), following the SARS outbreak, the Taipei City Government initiated a new public health plan using an integrated infection control system against emerging infectious diseases (EID). This new system integrated early detection of outbreaks (particularly in hospitals and schools), epidemiological investigation, and epidemiologically based public health prevention and control policies. The renovated Division of Disease Control and Prevention (Taipei’s CDC) also became the core operational unit for implementing crisis management procedures and facilitating policy. These systematic upgrades allowed Taipei’s CDC to quickly implement its Multi-Channel Mass Risk Communication Program during the 2007 outbreak of Acute Hemorrhagic Conjunctivitis. As reported by Hanvoravongchai et al (2010) the integration of national leadership into public health emergency preparedness planning and response activities in response to pandemic influenza can be seen through the participation of presidents or prime ministers on pandemic preparedness committees. In well-established health systems, pandemic preparedness is integrated within existing mechanisms, notably within the national disaster preparedness framework. In countries with a weak healthcare system, vertical programs are established to manage and coordinate pandemic preparedness and response.	Yen (2009) Hanvoravongchai (2010)	Moderate (Evidence from both studies was individually evaluated to be of moderate confidence level, with minor concerns on methodology and adequacy of data)
**Organizational Proximity of ERC Practitioners to National Health Leadership Response**	Organizational factors and restrictions need to be modified to facilitate timely release of information. Cope et al (2014) noted that a prominent issue in the People’s Republic of China was the inability of certain departments to release information, a restriction that was noted across departments and throughout the chain of command, from the local level through to the provincial and the national levels. The authors recommend increasing the freedom each department has to release relevant information, which has been reasonably verified, as a way to improve the system. The broader issue regarding restrictions on how information can be released directly ties into concerns about the timely release of information. Chambers et al (2012) described how during pandemic influenza in the UK, the only formal communication channel ‘up the chain of command’ was indirect, run through the regional Health Protection Agency (HPA) (Chambers et al 2012). The failure to allow information from the frontline to feed up the channels to national level decision making presented an issue. The UK relied on the Health Protection Agency, a quasi-independent agency, to lead, coordinate and manage the operational response at the local level—a role for which it was ill-equipped, given that its main mission is to provide disease surveillance and epidemiological advice at the national and local levels.	Cope (2014) Chambers (2012)	Moderate (Evidence from both studies was individually evaluated to be of moderate confidence level, with minor concerns on methodology and adequacy of data)
**Development of Laws, Regulations, Policies & Frameworks in Support of ERC Efforts**	Laws, regulations and frameworks contribute to define the context in which ERC functions and strategies are implemented. In some circumstances amendments may be necessary to facilitate such functions. Yen et al (2009) report on Taipei’s ability to launch a large-scale SMS campaign as a direct result of Taiwan’s Communicable Disease Act (2006). This act allowed government officials to override the people’s right to privacy when responding to epidemic disasters. In this case, the Taipei city government held a contract with Taiwan’s six major mobile phone companies, which committed them to six free public service messages (per year) to be sent to their users, if deemed necessary by the proper authorities. Lam et al (2008) describe an important achievement of the Hong Kong Special Administrative Region Government (HKSARG) when, in 2005, they signed a Cooperation Agreement on Response Mechanism for Public Health Emergencies with the Mainland Ministry of Health and Macao Health Bureau. Under this Cooperation Agreement, when a cross-boundary public health emergency occurs, the Mainland, Hong Kong and Macao forms a joint emergency response group to facilitate sharing of intelligence and expertise. Cope et al (2014) recommend the need for amendments to the infectious diseases prevention and control law of the People’s Republic of China to authorize more freedom for provincial and local public health agencies to release information. This would enhance the ability of local public health agencies to release information and improve risk communications, thereby addressing the barrier in the release of critical health information, reported by many agencies. Ikeda et al (2011) describe a framework of disaster risk governance presented as an implementation strategy for integrated risk management that incorporates innovative local coping capabilities that reduce disaster vulnerability. The framework is supported by a societal platform of disaster risk information called DRIP, developed in 2006 by the National Research Institute for Earth Science and Disaster Prevention in Japan as a tool for promoting improved disaster risk governance. DRIP is a societal platform of disaster risk information that works as a clearinghouse, collecting and disseminating scientific expertise on risk information from various disaster prevention organizations, fire brigades, and research institutions.	Yen (2009) Lam (2008) Cope (2014) Ikeda (2011)	Moderate (Evidence from three studies was individually evaluated to be of moderate confidence level, with minor concerns on methodology (Yen, Cope), adequacy of data (Yen, Ikeda) and coherence (Cope). Evidence from one study (Lam) was evaluated to be of low confidence level with significant concerns on methodology and adequacy of data)
**Emergency Risk Communication Training/ Exercises to enhance ERC integration**	Exercises and training can be conducted to identify barriers and successes in the integration of ERC functions into national and international public health emergency preparedness planning and response. For example, after the Hong Kong Special Administrative Region Government (HKSARG) updated their pandemic preparedness plan, they took part in the 2006 APEC Pandemic Response Exercise, which tested communications channels to ensure preparedness across Asia-Pacific Economic Cooperation economies in response to emergencies (Lam et al 2008). Hanvoravongchai et al (2010) describes a number of simulation exercises, mostly tabletop exercises, where officers discuss and manage a hypothetical pandemic situation in a round-table manner. For example, Thailand had at least one table-top exercise at both the central level and in each province. Vietnam has conducted many simulations for AHI preparedness at national, provincial and district levels, as well as at airports and borders. There were also a few regional (multi-country) table-top exercises coordinated by the World Health Organization, and one table-top exercise by the Mekong Basin Disease Surveillance Network (MBDS). Only Indonesia and Taiwan performed full-scale exercises involving real field activities. Indonesia's full-scale exercise, in Bali during April, 2008, was the first of its kind in the world. Most exercises reveal that management and coordination between various players, including non-health sector players, constitutes a major weakness in preparedness.	Lam (2008) Hanvoravongchai et al (2010)	Moderate (Evidence from both studies was individually evaluated to be of moderate confidence level, with minor concerns on methodology and adequacy of data)

**Table 5:** Synthesis of findings across methodological streams–Question 1, Chinese/ Mandarin literature.

Thematic area of practice	Synthesis of findings	Citations	CERQual assessment of confidence in evidence
**Utilization of Government Service “Weibo”, for ERC**	These studies discuss how most Chinese government agencies use the micro blog “Weibo,” Chinese twitter, for public communication during normal times and public health emergencies such as H1N1, H7N9, terrorist attacks, and the recent Ya’An earthquake, with the goals of achieving timely, open, two-way communications, monitoring public opinion, controlling rumors, addressing public concern, and improving government services. To effectively control online rumors, swift response is needed by releasing official counter-messages and by working with the police to identify and penalize the sources of rumors, and by assessing the effectiveness of these countermeasures by continuing monitoring Weibo. There might be potential for social media surveillance to be incorporated into mainstream disease surveillance and response systems. There has been an ongoing national effort to expand the use of government Weibo across the country and to establish a standardized operation, training and evaluation mechanism for Chinese government’s Weibo use and service performance. Since 2012, People's Public Opinion Monitoring Office and Weibo Data Center have routinely published performance evaluation and best practices on official microblogs (Weibo) of all levels of the Chinese government. Guidelines and regulations on government micro blogging have been developed by central and local governments and service providers (e.g. Government Micro blogging Operation Guidelines] by Sina Corporation, and “Autonomous Region Government Guidelines on Micro blog Use and Management” by XinJing.) Reports on government micro blog use and case studies on lessons learned and best practices have been developed and are disseminated on a biannual basis.	Chen et al, 2013; Chen et al, 2014; Liu et al., 2013; The People’s Public Opinion Monitoring Office and Weibo Data Center, 2012–2016; Zeng et al, 2015; Zhang XE et al, 2015; Zhou et al, 2015	Moderate (Limited details on survey items, process, and analysis)
**Collaboration between the US and Chinese CDCs**	These studies examine collaboration between the US CDC and China’s CDC to enhance the China’s efforts towards building ERC capacity and integrating ERC into public health emergency preparedness activities. China’s CDC has conducted assessments of ERC capacity and needs at local public health agencies, and identified barriers and possible solutions. Training materials have been created and tested among local CDC staff. It was found that there was a demonstrated need to develop an official ERC guideline by conducting needs assessments and reviews of the laws and regulation that guide the work of ERC (Hao, 2009, Ma, 2010; Zhang, 2011). Chinese public health professionals have been developing and revising the ERC guidelines and integrating them into current emergency preparedness plans.	Hao et al, 2009; Ma, 2010; Shao et al, 2014; Song et al, 2016; Xie et al, 2011; Zhang et al, 2011	Moderate (Limited details on survey items, process, and analysis)
**Integration of national health hotline into emergency preparedness system**	These studies look at 12320, the only official health hotline in China set up by China’s MoH. 12320 has been integrated into the emergency response system and tested as an important channel of communication during the 2008 formula contamination crisis, the 2008 Beijing Olympics, the 2009 A(H1N1) pandemic, and the recent measles campaign. As a direct two-way communication channel between the government and the public, 12320 was considered a trusted channel of communication by the public. It offered health consultation directly to the public, acted as an important emergency risk communications agent and had taken on the role of gathering public reaction data. It has been highly valued by China’s MoH and helped shape the ERC strategies. Due to its ease of access and two-way communication, 12320 played an integral role in ERC and public opinions monitoring during the A (H1N1) pandemic and the recent measles vaccination campaigns. Since then, efforts are being made to integrate 12320 into the existing public health response system and to develop a protocol to monitor future public reactions/opinions during routine public health activities and public health emergencies. 12320 provides services including infectious disease prevention and control, health care consultations and public health legal consultations in China’s provinces, municipalities and autonomous regions.	Jiang et al, 2012; Wang et al, 2010; Zhang XE et al, 2015	Moderate (Limited details on survey items, process, and analysis)

**Table 6:** Synthesis of findings across methodological streams–Questions 2 & 3 (combined).

Thematic area of practice	Synthesis of findings	Citations	CERQual assessment of confidence in evidence
**Creation of task forces/committees to enhance ERC**	The creation of task forces and committees with key stakeholders has been described as a mechanism for improving or facilitating information sharing between national and sub-national authorities and between agencies. The creation of a bioterrorism task force at the county level in New Jersey was described by Chess (2007) as an important mechanism for enhancing risk communications and trust among representatives of different agencies during the 2001 Anthrax incidents. Clarke and Chess (2006) describe the utilization of an Emergency Operations Committee (EOC) during a university’s response to the Anthrax incidents. In addition a number of components for the effective operation of networks, taskforces, and committees were identified. These include: the importance of existing relationships between responders prior to an incident (Nowell 2015), the role of network teams, as opposed to hierarchical teams, for improved decision making (Schraagen 2010), and the importance of information exchange and distribution between decision-making units (Bharosa 2010). They also include the importance of a Public Information Officer (PIO) for improved information dissemination (Howard 2012), and the role media should play for effective communication in an emerging infectious disease outbreak (Holmes 2009). Schraagen et al (2010) hypothesized that network teams work faster and arrive at more correct decisions than hierarchical teams Network structures. They allow teams to exchange information quickly, monitor each other’s performance, and build mutual trust. Network teams were found to perform faster than hierarchical teams, while maintaining the same level of accuracy in relatively simple environments. In relatively complex environments, network teams arrive at correct decisions more frequently than hierarchical teams. Holmes et al (2009) describe the importance of engaging media representatives immediately in discussions about potential emerging infectious disease outbreaks, including the role media should play and how the public health community can help them fulfill that role.	Chess (2007) Clarke (2006) Nowell (2015) Schraagen (2010) Bharosa (2010) Howard (2012) Holmes (2009)	Moderate (Evidence from four different studies (Clarke, Schraagen, Bharosa 2010 and Holmes) was individually evaluated to be of moderate confidence level with minor concerns on methodology and adequacy of data. Evidence from remaining three studies (Chess, Nowell, Howard) was evaluated to be of low confidence level (methodology/ data concerns), but the same evidence is reinforced by the other four studies.)
**Networks to enhance information sharing**	Regional disease surveillance networks may provide a useful mechanism for information sharing. Gresham et al. describes the Middle East Consortium on Infectious Disease Surveillance (MECIDS), a regional disease surveillance network of public health experts and ministry of health officials from Israel, the Palestinian Authority, and Jordan. MECIDS unites public health officials of differing Middle Eastern nationalities and contributes to regional health and stability by engaging in regular cross-border information exchange, conducting regular executive board meetings, performing laboratory and risk communications training, and implementing innovative communication technology. During H1N1, the MECIDS partners agreed to prompt and coordinated border and airport screening, laboratory testing, information exchange, and common communication strategies. This coordination can be largely contributed to the existence of both trust and well-exercised national and regional pandemic preparedness plans, which were initially established within this network. Moore and Dausey describes the Mekong Basin Disease Surveillance (MBDS), a cooperating network of six countries to collaborate on sub-regional infectious disease surveillance and control—Cambodia, China (originally just Yunnan province and, since 2008, Guangxi Province as well), Lao People’s Democratic Republic, Myanmar, Thailand and Vietnam. During the H1N1 pandemic, MBDS health leaders perceived their pandemic responses to be effective in areas that, prior to the creation of the network, were considered problematic. Participants noted the ability of their country level surveillance systems to exchange information efficiently within the country and the importance of MBDS for enabling timely coordinated regional response to detect disease at cross border sites and prevent the spread of the virus across countries.	Gresham (2009) Moore (2011)	Moderate (Evidence from both studies was individually evaluated to be of moderate confidence level with minor concerns on methodology and/or adequacy of data)
**Use of information systems to enhance ERC**	Tools and platforms can be used as information systems to share information. Celik et al. (2010) describes enhancements in the use of the communication infrastructure by comparing the response to two earthquakes that occurred in Turkey, and underlines the importance of organizational learning, as well as investments in information technology, to enhance capacity for the search for, acquisition and exchange of information. Seyedin et al. (2011) describes the types of databases and information system in Iran should include for effective communications in emergency management. Ipe et al. (2010) describes a surveillance system used in the US, named the Medical Electronic Surveillance and Intelligence System (MEDSIS), and analyzes the role of stakeholders in the exchange of information through this system. Bharosa et al (2009) reviewed the flood management system, named Flood Information and Warning System (FLIWAS), for improved Dutch-German cooperation in flood scenarios. FLIWAS was developed to optimize the exchange of information during threatening high water situations within and between water management and calamity management organizations. Leonard et al. (2014) describes different mechanisms of communication in relation to volcanic eruptions in New Zealand abased on the audience (i.e. scientists, emergency managers, media, public, etc.). Thiago et al. (2013) describes the role of social networks and mobile phones in alerting and preparing people to avoid or face natural disasters in a region of Brazil. Collaboration between government and Civil Defense in rapidly testing and developing new channels of communication was found to be successful in terms of quantity and quality of information shared both within the organization and with the target population. Based on survey results of a sample of first responders for a disastrous typhoon in Taiwan in 2009, Chang and Wang (2013) present the official information sharing and coordination operations and an emergency management information system (EMIS) in Taiwan. The authors also discuss what needs to be done, or is currently being done, to improve the system. Kapucu et al. (2006) describes how the lack of an integrated information system greatly limited the coordinated response of agencies (police, fire department and port authority) following the World Trade Center terrorist attack in New York city. In contrast to the more technical operating platforms, Militello et al (2007) suggests that low cost, paper based tools, including notebooks, whiteboards, and telephone books, provide important tools for communication and can support asymmetric knowledge in an Emergency Operations Center (EOC). Often times, EOC teams are made up of individuals with differing levels of experience in regards to crisis management, familiarity with emergency response tools and procedures, which can make it difficult to effectively utilize electronic tools without onsite support.	Celik (2010) Seyedin (2011) Ipe (2010) Bharosa (2009) Leonard (2014) Thiago (2013) Chang (2013) Kapucu (2006) Militello (2007)	Moderate (Evidence from seven different studies (Ipe, Bharosa 2009, Leonard, Thiago, Kapucu, Militello and Chang) was individually evaluated to be of moderate confidence level with minor concerns on methodology and/or adequacy of data. Evidence from remaining two studies (Celik from Turkey and Seyedin from Iran) was evaluated to be of low confidence level (adequacy of data concerns).)
**Local stakeholders’ engagement**	Engagement of local stakeholders is important for the effectiveness of the ERC strategy. The literature presents examples of mechanisms for the engagement of local stakeholders in communication efforts. Ardalan et al. (2009) describe the use of Village Disaster Taskforces, which act as operational units in an early warning mechanism for flooding in Iran. Cole et al. (2014) describes the importance of using existing social networks in small municipalities for disaster risk reduction activities. Shepherd et al. (2014) addresses the issue of communicating with culturally diverse communities, and the need for centralizing resources that can be used to facilitate communication with these groups. Gultom et al. (2014) describes the use of a community based communication system in Indonesia to facilitate the response to volcanic eruptions. Mulyasari et al. (2013) describes the role of networks of women in facilitating communication for all hazards in Indonesia. Lei (2015) describes the barriers to effective information sharing and response coordination among agencies during a highway emergency and proposes possible solutions.	Ardalan (2009) Cole (2014) Shepherd (2014) Gultom (2014) Mulyasari (2013) Lei (2015)	Moderate (Evidence from five different studies (Ardalan, Cole, Shepherd, Gultom, Lei) was individually evaluated to be of moderate confidence level with minor concerns on adequacy of data. Evidence from one study (Mulyasari) was evaluated to be of low confidence level (method and data concerns).)

For the overall conduct of this systematic review, the PRISMA guidelines were followed to the extent possible given the limitations inherent to a review largely based on qualitative studies [15].

## Results

### Characterization of the literature

**Fig 1:**
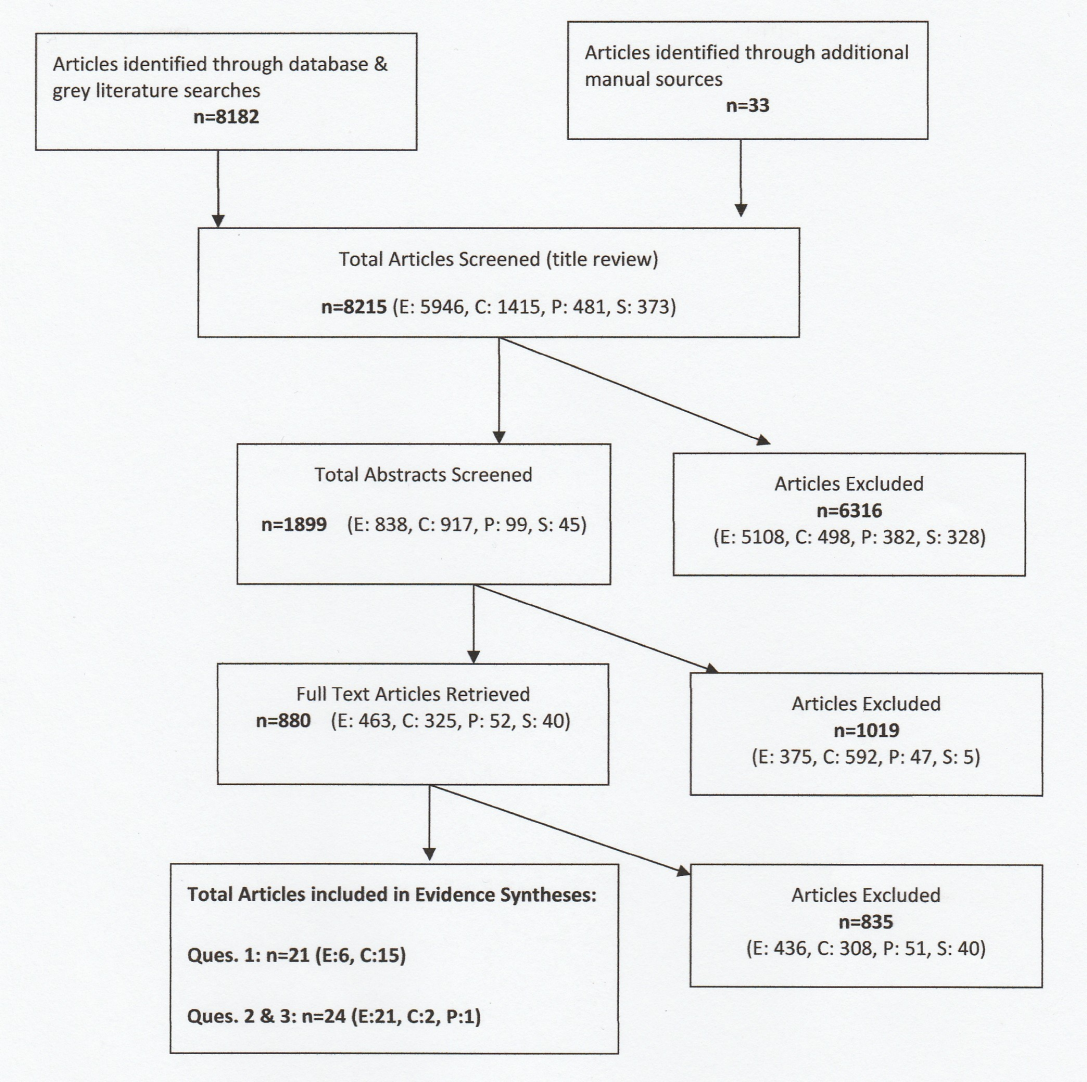
The flow of literature [adapted from the PRISMA flow diagram 2009]. E = English, C = Chinese, P = Portuguese, S = Spanish.

Among the 8,215 articles retrieved, 5,946 were in English (may have included other language publications that had English abstracts), 1,415 in Chinese, 481 in Portuguese and 373 in Spanish. Through title screening, 6,316 (77%) articles were excluded, while 1,899 (23%) abstracts were reviewed, leading to 880 (11% of all retrieved) full text articles for further scrutiny. After the final stage appraisal, 21 full text articles were selected for Question 1 (6 in English and 15 in Chinese), and 24 more for Questions 2 and 3 combined (21 in English, 2 in Chinese and 1 in Portuguese). Fig 1 depicts the step-wise flow of literature.

Among the 21 articles identified as related to question (1), 6 (28.5%) were qualitative, 6 mixed-methods, 5 (24%) quantitative and 4 (19%) case studies. Most articles focused on ERC in China, Hong Kong and Taiwan (18, 86%), while the others covered ERC in Cambodia, Indonesia, Japan, Laos, Vietnam, Thailand and UK (1 each, with overlap). Twelve (57%) of these 21 articles addressed all hazard situations (general disasters), while 9 were based on pandemic influenza and other infectious disease outbreaks. Tables 4 and 5 summarize the evidence. S1 and S2 Tables list the individual study findings within methodological streams and evaluation of confidence.

Among the 24 articles included for the final evidence syntheses for questions (2) and (3) combined, the majority (12, 50%) were qualitative in design, with 5 (21%) case studies, 4 (17%) quantitative and 3 (12%) mixed method approaches. Seven (29%) articles focused on the US, and 3 each on Netherlands and China (including Hong Kong and Taiwan). Other regions represented included Asia (Cambodia, Vietnam, Thailand, Indonesia, Laos, and Myanmar), the Middle East (Turkey, Iran, Israel, Palestine, and Jordan), Australia, New Zealand, Canada and Brazil. Among the disaster-types, 7 (29%) articles addressed all-hazard situations, 4 addressed pandemic influenza/infectious diseases, 3 described floods, 2 each addressed hurricanes/ tornados, volcanos, terrorism and anthrax scares, and 1 each described a wildfire, earthquake or other major accident. Table 6 summarizes the evidence. S3 Table lists the individual study findings within methodological streams and evaluation of confidence.

**Evidence synthesis: (1)** How can ERC best be integrated into national and international public health emergency preparedness planning and response activities?

### English language literature

The articles identified as relevant to this question presented examples of mechanisms that might lead to the integration of ERC functions into the leadership structure. Such mechanisms were summarized under four themes: (1) placing ERC functions into the national leadership structure [16, 17]; (2) creating organizational proximity of ERC practitioners to national response leadership [18, 19]; (3) developing laws, regulations, policies, and frameworks in support of ERC [16, 18, 20, 21]; and (4) the use of trainings and exercises as a mechanism for testing the effectiveness of the system [17, 20] (cross cutting theme).

Restructuring of existing organizational components of agencies engaged in preparedness and response efforts, as well as developing novel intra- and inter-agency coordination frameworks were often needed to achieve integration of ERC functions into the national preparedness/disaster response leadership. Yen et al. [16] described how a new public health policy by the Taipei City Government formed the basis for implementing an integrated infection control system to respond to emerging infectious diseases during the aftermath of the SARS outbreak in 2003. The Taipei Division of Disease Control and Prevention was reformed to function as the nodal unit for implementing crisis management programs and policies, including integration of early outbreak detection through hospital-based and school-based surveillance, prompt epidemiological investigations and preventive responses. These structural reforms allowed the Division to launch a swift response during the 2007 acute hemorrhagic conjunctivitis outbreak through its multi-channel, mass risk communication program. In the same context, it was Taiwan's Communicable Disease Act of 2006 which facilitated Taipei's ability to launch large-scale SMS campaigns in 2007, as this Act provided the necessary legal platform to allow government agencies to override people’s right to privacy (government could use cellular service providers to send out six free public service messages per year) when responding to epidemics or disasters.

Assessing pandemic influenza preparedness in six Asian countries (Cambodia, Indonesia, Laos, Taiwan, Thailand and Vietnam), Hanvoravongchai and colleagues [17] observed that in nations with well-functioning health systems, pandemic preparedness was integrated within existing mechanisms such as national disaster preparedness frameworks; while those with weaker systems relied heavily on vertical programs for coordination and response. The team further described the importance of simulation exercises in these countries that demonstrated the gaps in coordination between the various stakeholders (health sector and beyond). Thailand ensured at least one table-top exercise at the central level and in each province; Vietnam covered airports and borders, in addition to administrative levels; Indonesia conducted a first-of-its-kind full scale preparedness exercise in Bali in 2008. Furthermore, the WHO and the Mekong Basin Disease Surveillance (MBDS) Network coordinated several regional cross-country exercises.

Cope [18] noted that the lack of authority to release information to the public was a critical barrier to ERC throughout the the chain of command in the Chinese public health system. On the other hand, shared public health intelligence between the Hong Kong Special Administrative Regional Government, Mainland (China) Ministry of Health and Macao Health Bureau facilitated functioning of joint emergency responses in the event of cross-boundary public health emergencies. [20] DRIP, a societal platform for disaster risk information which facilitated the acquisition and dissemination of scientific expertise on risk information from a large number of governmental/non-governmental agencies and research institutions, was utilized as a major tool in Japan's disaster risk governance. [21]

#### Quality assessment

For each of these thematic areas, the overall synthesized evidence was considered to be of moderate confidence level (GRADE-CERQual). Most of the contributory publications (to each area) were individually judged to have only minor concerns regarding methodology, and/or adequacy of data, and/or coherence; hence these articles individually provided evidence of a "moderate" confidence level. The confidence in the pooled evidence is a reflection of such a majority of articles.

### Chinese language literature

The articles identified to respond to question (1) described practices across three functional areas: (1) how various government agencies used the micro-blogging platform "Weibo" for ERC [22–28]; (2) integration of a national health hotline into the emergency preparedness and management system [27, 29, 30]; and (3) collaboration between the US and Chinese CDCs to build ERC capacity across the country [31–36].

Government agencies in China utilized the social media platform "Weibo" (biggest Chinese micro-blogging platform, often referred to as “Chinese Twitter”) for public communication during normal times as well as in public health emergencies (like H1N1, H7N9, terrorist attacks, and the Ya’An earthquake) with the goals of achieving timely, open, two-way communications, monitoring public opinion, controlling rumors, addressing public concerns and improving government services. There had been a consistent national effort to expand the use of Weibo across China, and to establish a standardized operation, training and evaluation mechanism for its service performance. [22–28] In order to effectively control online rumors during health or humanitarian crises, a swift response was mounted by releasing official counter-messages, working with law enforcement to identify and penalize sources of rumors, and assessing the effectiveness of these counter-measures through continuous monitoring of Weibo.

A national hotline service (12320, China's only such call-in facility) was developed by the Ministry of Health (MoH) and integrated into the emergency response system. This was successfully tested as an important channel of communication during the 2008 formula contamination crisis, 2008 Beijing Olympics, 2009 A(H1N1) pandemic, and the recent measles campaign. [27, 29, 30] This hotline has helped shape national ERC strategies such as developing protocols to monitor public reactions and opinions through providing a direct, two-way communication between health agencies and the public. and has facilitated public health consultations across China’s provinces, municipalities and autonomous regions.

Within China's governance framework, these novel approaches had helped integrating ERC as a system response involving multiple agencies and the target (affected) population. Further, the Chinese CDC had been working in close collaboration with its US counterpart to conduct assessment of ERC needs at local public health agencies, through conducting tabletop and functional exercises. [31–36]

#### Quality assessment

Similar to synthesized evidence from English language publications, the overall synthesized evidence was considered to be of moderate confidence level (CERQual) for each of these three functional areas.

**Evidence synthesis: (2)** What are the best mechanism(s) to establish effective intra-agency, inter-agency, and/or cross-jurisdictional (such as cross-border; national with sub-national jurisdictions, etc.) information sharing for emergency risk communication? **and (3)** What are the best practices and protocols to ensure coordination of risk communication activities between responding agencies across organizations and levels of response?

The articles (in English, Chinese, and Portuguese) identified to respond to these two questions (combined) presented examples of mechanisms to enhance information sharing and coordination. Such mechanisms were summarized under three themes: (1) creation of task forces/committees [37–43] and networks [44, 45] to enhance ERC, and their elements of functionality); (2) use of information systems to enhance ERC (tools and platforms) [46–54]; and (3) mechanisms to facilitate local stakeholders’ engagement in ERC [55–60].

The formation and functioning of collaborative platforms like task forces, networks and committees had been attributed to facilitate efficient information sharing between national and sub-national authorities, as well as between agencies. Chess et al. [37] cited the role of a bioterrorism task force in New Jersey, USA which served as a platform for ERC sharing between partner agencies like public health and law enforcement during to the 2001 Anthrax incidents. The diverse agencies had developed mutual trust through this pre-existing task force, and this lay the foundation for improved intersectoral coordination and intelligence exchange. It had been pointed out that emergency responders were far more likely to trust and interact with people/agencies with whom they had an existing professional relationship, and such networking improved agility in carrying out emergency response measures. [39, 40] Specific roles for Information Managers and/or Public Information Officers at local agencies had been proposed to be improve intra-agency coordination. [41, 42]

Gresham [44] described the collaborative health information sharing network, Middle East Consortium on Infectious Disease Surveillance, between Israel, Jordan and the Palestinian Authority. This forum brought together politically divergent states and served as a platform to boost regional health intelligence exchange, capacity development through laboratory and risk communications training, and implementation of innovative communication technology. This partnership greatly assisted cross-border preparedness (including airport and border screening, laboratory testing) and ERC strategies during the H1N1 pandemic, building on pre-existing trust and thoroughly exercised national/regional emergency plans and protocols. Similarly, the MBDS Network between Cambodia, China (originally just Yunnan province and, since 2008, including Guangxi Province), Lao People’s Democratic Republic, Myanmar, Thailand and Vietnam served as a platform to coordinate sub-regional infectious disease surveillance and control. Regional-level coordinated preparedness and prevention efforts led to better control of the pandemic within the participating countries. [45]

The literature provided several examples where planned investment in communication infrastructure including better operationalization of wireless communication channels, increased coordination between responding agencies, and better understanding of communication needs improved the overall disaster management. This was specifically demonstrated during the response to two earthquakes, three months apart, in Turkey. [46] Thiago et al [51] described the successful collaboration between the government and Civil Defense in Brazil which led to the development and rapid testing of novel channels of ERC utilizing social networks (Facebook, Twitter) and mobile phones. Funded by the European Union, the Flood Information and Warning System along the Dutch-German border had optimized communication between water-management and crisis-management agencies. [49] On the other hand, Kapucu [53] described how the absence of an integrated information system greatly hindered coordinated response of agencies (police, fire department and port authority) following the World Trade Center terrorist attack in New York City. Chang et al. [52] studied the process of information sharing and coordination within Taiwan's emergency management information system during the catastrophic 2009 typhoon. They recommended identification and designation of an agency that had the best geographic reach (in this case, the police) to lead the process of ERC sharing. Militello [54] had observed that, given the diverse levels of knowledge and experience of different teams at an emergency operations center (EOC), low-cost substitutes like notebooks, whiteboards, and telephone books, in addition to electronic tools, could greatly improve functional efficiency by removing any asymmetric skills barriers.

Different mechanisms were proposed to engage local stakeholders in formulating and implementing ERC strategies. Ardalan [55] suggested formation of Village Disaster Taskforces through community participation in Iran, to function as operational units in the early warning mechanism by facilitating spread of ERC to the lowest levels of the chain. Cole et al. [56] proposed that community emergency management coordinators should look to utilize existing social networks in small rural municipalities for public education and disaster risk reduction activities. Citing the example of 2011 Brisbane floods (Australia), Shepherd [57] emphasized the need to address culturally and linguistically diverse populations through the incorporation of appropriate ERC materials into centralized resources. Gultom [58] described a community-based risk information sharing network in Indonesia, the Merapi Circle Information Networks, which developed local radio stations and recruited trusted community representatives to harness ground resources in order to be better equipped in emergency preparedness. A study from Badung, Indonesia found that the Women's Welfare Association leaders were in a unique position to act as key facilitators in the early warning system at sub-district, city or ward levels of governance. [59]

#### Quality assessment

Similar to the evidence syntheses for Question 1, for each of these thematic areas the overall synthesized evidence was considered to be of moderate confidence level (CERQual).

## Discussion

The functioning of ERC is intricately linked to the varying political and cultural landscapes present across nations. Therefore, in some circumstances, centralized ERC systems may work better than localized ones, or vice versa. Researchers had noted that decentralized health systems (e.g. as in Indonesia) faced greater challenges in implementing preventive and outbreak response measures, and the level of efficiency depended heavily on local political commitment. [17] In contrast, there was a need for increased decision-making power at the level of provincial and local public health agencies in China to enable them to release critical ERC to the public, circumventing barriers in organizational hierarchy. [18] Hence, issues like political goodwill and leadership, as well as the structure of the national health system (degree of centralization) are to be considered as key factors in planning and policy-making for ERC.

The development of ERC policies and capacities through regional partnerships and guidelines seemed to be well received. For example, the European Union (EU) had enacted legislation on a cross-border integrated emergency response system, including coordination and information exchange between constituent nations. The Health Security Committee (HSC) Communicators’ Network under the EU provides crisis communication expertise and guidance as part of a comprehensive strategy for the successful management of public health threats. The information-sharing protocol is implemented through the establishment and activation of a list of contact points within the EU, the European CDC and the WHO. [61]

Geographic variations in capacity and practice of ERC strongly necessitated the formulation of evidence-based universal guidelines by the WHO to help member states develop frameworks to integrate ERC as a system response during emergencies of public health concern. Such communication needs to be transparent, timely and based on the best available scientific evidence, in order to ensure the maximal physical, social and economic well-being of citizens.

The identified literature referred to mechanisms, practices from the field, and recommendations that were derived from planning or response efforts implemented at the national or local levels in specific countries, but did not provide direct evidence of transferability to other contexts. Factors that seemed to be related to the integration of ERC functions in national and international public health emergency preparedness, planning and response activities included renovation of components of the leadership structure when needed, modification of organizational factors, nullifying restrictions that might hinder the timely release of information, and amendments to laws and regulations where feasible. Exercises and trainings were recognized as strategies to identify barriers and successes in the integration of ERC functions into preparedness, planning and response efforts. Key elements to enhance information sharing and coordination across organizations included the creation of networks, task-forces and committees across disciplines, organizations and geographic areas. The functionality of information systems was a key element for the sharing of information by tailoring such systems to the needs of the users. Engagement of local stakeholders was equally important to guarantee the flow of information up and down the incident command system.

Despite conducting a very thorough literature review across multiple language databases, the authors felt that more sensitive approaches may be needed to gather useful evidence in a non-Western non-English context, for example, when conducting region-specific case studies. It was observed that the Chinese language articles tended to be succinct in the methodology section and elaborative about policy or program implications, with an emphasis on status report and actions taken or recommendations for future steps. This utility-driven approach might be useful in offering actionable information to practitioners on the ground in the context of China, but presented a challenge in quality assessment. With the Spanish and Portuguese language publications, there was a general paucity of empirical literature on ERC. Risk communication in emergency preparedness was mainly addressed by the social health, communication and technology, and human sciences fields; very little of this work was produced by researchers or practitioners working in public health. In addition, differences in organizational response structure, especially in Latin America, contributed to this overall finding.

On the whole, few empirical studies, especially from low- and middle-income countries were related to the WHO research questions. The authors attempted to circumvent this shortcoming by searching databases in Chinese, Portuguese and Spanish, as well as relaxing the strict definition for empirical literature to include more case studies and to reflect a broader distribution of country experiences and knowledge. However, this observed bias against empirical studies from low- and middle-income countries may be partly due to the limitations of the authors in their ability to assess a broader range of languages. Furthermore, the fact that ERC is still not precisely-defined as a field of research meant that there were challenges in identifying sensitive search terms and keywords that would incorporate the varied disciplines that cover this field. It is probable that in addition to differences in terms across disciplines, terms may also differ across nations, and even among professionals trained in similar disciplines.

The authors believe that the lack of empirical studies across the questions solicited by the WHO demonstrates an overall need for research in these areas. However, an accurate identification of research gaps should be achieved by integrating the results of this review with case studies across the WHO regions to better understand what type of evidence is needed in practice across the multitude of ERC functions. Such an approach may ensure that research is produced in the topic areas of greatest need for practice.

## Supporting information

S1 Table Individual study findings within methodological streams and evaluation of confidence–Question 1, English language literature. (PDF)

S2 Table Individual study findings within methodological streams and evaluation of confidence–Question 1, Chinese/ Mandarin literature. (PDF)

S3 Table Individual study findings within methodological streams and evaluation of confidence–Questions 2 & 3 (combined). (PDF)

S4 Table PRISMA 2009 checklist. (DOCX)
