# Supplementary material for: Integrating emergency risk communication (ERC) into the public health system response: Systematic review of literature to aid formulation of the 2017 WHO Guideline for ERC policy and practice
Source: PLoS One. 2018 Oct 31;13(10):e0205555. doi: 10.1371/journal.pone.0205555 (PMC6209198; doi:10.1371/journal.pone.0205555)
Supplement: S1 Table — (PDF) [file pone.0205555.s001.pdf]

S1 Table: Individual study findings within methodological streams and evaluation of confidence – Question 1, English language literature

Thematic area of practice	Citation	Type of Emergency<br>Study Methods<br>Country/Geographic Context	Summary findings	CERQual assessment of confidence in the evidence
**Placement of ERC Functions in National Leadership Structure**	Yen M-Y, Wu T-SJ, Chiu AW-H, Wong W-W, Wang P-E, et al. Taipei's Use of a Multi-Channel Mass Risk Communication Program to Rapidly Reverse an Epidemic of Highly Communicable Disease. *PLoS ONE.* 2009. 4(11): e7962. doi:10.1371/journal.pone.0007962	Acute Hemorrhagic Conjunctivitis (AHC)<br><br>Case Study Approach - Survey<br><br>Taiwan	The integration of ERC in national public health emergency planning and response activities can be supported by the renovation of components of the leadership structure when needed. The study from Yen et al (2009) describes how, following the SARS outbreak, the Taipei City Government initiated a new public health plan using an integrated infection control system against emerging infectious diseases (EID). This new system integrated early detection of outbreaks (particularly in hospitals and schools), epidemiological investigation, and epidemiologically based public health prevention and control policies. The renovated Division of Disease Control and Prevention (Taipei's CDC) also became the core operational unit for implementing crisis management procedures and facilitating policy. These systematic upgrades allowed for quick enactment of multi-channel risk communication measures during the 2007 outbreak of conjunctivitis. Rather than rely on the media alone to convey productive messages regarding the epidemic, Taipei's CDC was able to implement its Multi-Channel Mass Risk Communication Program to reach the public directly during critical points in the epidemic.	Moderate<br><br>(Minor concerns on methodology and adequacy of data)
	Hanvoravongchai P., Adisasmito W., Chau PN., Alexandra Conseil A., de Sa J., Krumkamp R., Mounier-Jack S., Phommasack B., Putthasri W., Shih CS., Touch S., Coker R. for the AsiaFluCap project. Pandemic influenza preparedness and health systems challenges in Asia: results from rapid analyses in 6 Asian countries. *BMC Public Health*. 2010. 10:322	Pandemic Influenza<br><br>Mixed Method Systematic Rapid Assessment analytical framework: Review of published data and key informant interviews<br><br>Cambodia Indonesia Laos PDR Taiwan Thailand Vietnam	The integration of ERC functions in national leadership structures can be achieved by the creation of preparedness committees and frameworks within each country political context. Hanvoravongchai et al (2010) presents the finding of a rapid assessment of pandemic preparedness in six countries. In all countries, the pandemic preparedness committees were headed by the president or prime minister or his/her representative. In well-established health systems, pandemic preparedness is integrated within existing mechanisms, notably within the national disaster preparedness framework. In countries with a weak healthcare system, new vertical programs had to be established to manage and coordinate pandemic preparedness and response. The nature of pandemic governance also depends on the existing political context. Decentralized countries have greater challenges to deal with during both outbreaks and pandemics. In a decentralized system, like in Indonesia, the level of political commitment may affect the level of investment in pandemic preparedness.	Moderate<br><br>(Minor concerns on methodology and adequacy of data)

Thematic area of practice	Citation	Type of Emergency<br>Study Methods<br>Country/Geographic Context	Summary findings	CERQual assessment of confidence in the evidence
**Organizational Proximity of ERC Practitioners to National Health Leadership Response**	Cope JR., Frost M., Richun L., Xie R. Assessing Knowledge and Application of Emergency Risk Communication Principles Among Public Health Workers in<br><br>China. *Disaster Med Public Health Prep*. 2014 June. 8(3): 199–205.	General Disasters<br><br>Multi-province survey of public health officials & interviews<br><br>China	Actions should be taken to enhance the timely release of information for example by increasing the authority of various departments in releasing relevant information that has been reasonably verified. Cope et al (2014) presented findings from a survey and interviews conducted with public health workers in China. A prominent issue detected by the authors was the inability of some departments to release information. This restriction was noted across departments, as well as through the chain of command, starting at the local level through to the provincial and national levels.	Moderate<br><br>(Minor concerns on methodology and adequacy of data)
	Chambers J., Barker K., Rouse A. Reflections on the UK's approach to the 2009 swine flu pandemic: Conflicts between national government and the local management of the public<br><br>health response. *Health & Place*. 2012. 18: 737-745.	Pandemic Influenza<br><br>Case study<br><br>UK	Organizational proximity of ERC practitioners and functions to the national level leadership can be achieved by enacting responsive multi-level communication mechanisms and by harnessing local public health expertise. Chambers et al (2012) describe that, during the response to pandemic influenza in the UK, the only formal channel of communication 'up the chain of command' was indirect, through the regional Health Protection Agency (HPA). The UK relied on HPA, to lead, coordinate and manage the operational response at the local level—a role for which it was ill-equipped, given that its main mission is to provide disease surveillance and epidemiological advice at the national and local levels.	Moderate<br><br>(Minor concerns on methodology)
**Development of Laws, Regulations, Policies & Frameworks in Support of ERC Efforts**	Yen M-Y, Wu T-SJ, Chiu AW-H, Wong W-W, Wang P-E, et al. Taipei's Use of a Multi-Channel Mass Risk Communication Program to Rapidly Reverse an Epidemic of Highly Communicable Disease. *PLoS ONE.* 2009. 4(11): e7962. doi:10.1371/journal.pone.0007962	Acute Hemorrhagic Conjunctivitis (AHC)<br><br>Case Study Approach - Survey<br><br>Taiwan	The integration of ERC in national and international public health emergency preparedness planning and response activities needs to be contextualized to existing laws and regulation. This article discusses how Taiwan's Communicable Disease Act (2006) allowed Taipei to launch a large-scale SMS campaign. This act allowed government officials to override the people's right to privacy when responding to epidemic disasters. In this case, the Taipei city government held a contract with Taiwan's six major mobile phone companies, which committed them to six free public service messages (per year) to be sent to their users, if deemed necessary by the proper authorities. The SMS message, in response to the Acute Hemorrhagic Conjunctivitis (AHC) epidemic, was sent to 2.2 million registered mobile phone users in Taipei City.	Moderate<br><br>(Minor concerns on methodology and adequacy of data)

Thematic area of practice	Citation	Type of Emergency<br>Study Methods<br>Country/Geographic Context	Summary findings	CERQual assessment of confidence in the evidence
**Development of Laws, Regulations, Policies & Frameworks in Support of ERC Efforts**<br><br>*(continued)*	Lam PY. Avian Influenza and Pandemic Influenza Preparedness in Hong Kong. *Ann Acad Med Singapore.* 2008. 37:489-9	Pandemic Influenza<br><br>Case Study<br><br>Hong Kong	Sharing of intelligence and expertise can be achieved by the creation of agreements between countries for a better integration of ERC in international, cross-country planning and response efforts as. Lam et al (2008) describe the role of the Cooperation Agreement on Response Mechanism for Public Health Emergencies, signed by the Hong Kong Special Administrative Region Government (HKSARG), the Mainland Ministry of Health and the Macao Health Bureau. Under this Cooperation Agreement, when a cross-boundary public health emergency occurs, the Mainland, Hong Kong and Macao will form a joint emergency response group to facilitate sharing of intelligence and expertise.	Low<br><br>(Significant concerns on methodology and adequacy of data)
	Cope JR., Frost M., Richun L., Xie R. Assessing Knowledge and Application of Emergency Risk<br><br>Communication Principles Among Public Health Workers in<br><br>China. *Disaster Med Public Health Prep*. 2014 June. 8(3): 199–205.	General Disasters<br><br>Multi-province survey of public health officials & interviews<br><br>China	The creation of amendments to existing laws may facilitate a better integration of ERC in the response. This article recommends that amendments be made to the People's Republic of China infectious diseases prevention and control law to authorize provincial and local public health agencies more freedom to release information. This will allow for improvements in the response time to public health emergencies by allowing agencies the freedom to readily inform their populations with critical health information.<br><br>The authors also suggest that all public health agencies should be required to develop a risk communication operational plan, which would provide a template from which dedicated risk communicators can work.	Moderate<br><br>(Minor concerns on methodology and coherence)
	Ikeda S., Nagasaka T. An Emergent Framework of Disaster Risk Governance towards Innovating Coping Capability for Reducing Disaster Risks in Local Communities. *Int. J. Disaster Risk Sci.* 2011. 2(2): 1–9.	General Disasters<br><br>Case Study<br><br>Japan	DRIP is a promising societal platform of disaster risk information that works as a clearinghouse, collecting and disseminating scientific expertise on risk information from various disaster prevention organizations, fire brigades, and research institutions. DRIP also communicates local and experiential information from residents and civic organizations under interoperable environments linked by multilayered networks. Ikeda et al discuss this emergent framework of disaster risk governance, presented as an implementation strategy for integrated risk management that incorporates innovative local coping capabilities that reduce disaster vulnerability. The framework is supported by a societal platform of disaster risk information, called DRIP, which National Research Institute for Earth Science and Disaster Prevention, Japan has developed since 2006 as a tool for promoting improved disaster risk governance.	Moderate<br><br>(Minor concerns on adequacy of data)

Thematic area of practice	Citation	Type of Emergency<br>Study Methods<br>Country/Geographic Context	Summary findings	CERQual assessment of confidence in the evidence
**Emergency Risk Communication Training/ Exercises to enhance ERC integration**	Lam PY. Avian Influenza and Pandemic Influenza Preparedness in Hong Kong. *Ann Acad Med Singapore.* 2008. 37:489-9	Pandemic Influenza<br><br>Case Study<br><br>Hong Kong	Exercises can be used to test ERC functions across countries. Lam et al (2008) discuss how the Hong Kong Special Administrative Region Government (HKSARG) took part in the APEC Pandemic Response Exercise 2006, which tested communications channels for ensuring preparedness across Asia-Pacific Economic Cooperation economies in responding to emergencies.	Moderate<br><br>(Minor concerns on methodology and adequacy of data)
	Hanvoravongchai P., Adisasmito W., Chau PN., Alexandra Conseil A., de Sa J., Krumkamp R., Mounier-Jack S., Phommasack B., Putthasri W., Shih CS., Touch S., Coker R. for the AsiaFluCap project. Pandemic influenza preparedness and health<br><br>systems challenges in Asia: results from rapid analyses in 6 Asian countries. *BMC Public Health*. 2010. 10:322	Pandemic Influenza<br><br>Mixed Method -Systematic Rapid Assessment analytical framework: Review of published data and KI interviews<br><br>Cambodia Indonesia Lao PDR Taiwan Thailand Vietnam	Exercises can be used to test ERC functions across countries. Hanvoravongchai et al (2010) note that a number of simulation exercises have been conducted in all six countries it investigated. Most of the exercises were table-top style, where relevant officers discuss and manage a hypothetical pandemic situation in a round-table manner. For example, Thailand had at least one table-top exercise at both the central level and in each province. Vietnam has conducted many simulations for AHI preparedness at the national, provincial and district levels, as well as at airport and borders. There were also a few regional (multi-country) table-top exercises coordinated by the World Health Organization and one table-top exercise by the Mekong Basin Disease Surveillance Network (MBDS). Only Indonesia and Taiwan had full-scale exercises involving real field activities. Indonesia's full-scale exercise, in Bali during April, 2008, was the first of its kind in the world. Most exercises reveal that management and coordination between various players, including non-health sector players, constitutes a major weakness in preparedness.	Moderate<br><br>(Minor concerns on adequacy of data)
