# Supplementary material for: Integrating emergency risk communication (ERC) into the public health system response: Systematic review of literature to aid formulation of the 2017 WHO Guideline for ERC policy and practice
Source: PLoS One. 2018 Oct 31;13(10):e0205555. doi: 10.1371/journal.pone.0205555 (PMC6209198; doi:10.1371/journal.pone.0205555)
Supplement: S2 Table — (PDF) [file pone.0205555.s002.pdf]

S2 Table: Individual study findings within methodological streams and evaluation of confidence – Question 1, Chinese/ Mandarin literature

Thematic area of practice	Citation	Type of Emergency Study Methods	Summary findings	CERQual assessment of confidence in the evidence
**Utilization of government controlled Chinese Twitter "Weibo" for ERC**	Chen et al (2013)	General Disasters<br><br>Qualitative-Content Analysis/ Governmental Weibo [Twitter]	Chinese Government Weibo [Twitter] has been a part of the standard emergency response mechanism. When using Weibo in conducting ERC, each responding agency should construct micro blog messages aligned with their own roles and responsibilities. Such clarity may help the public process information received and avoid confusion. It is also important for these agencies to assume the roles of information disseminator, the leader of conversation, and the monitor of public opinions for feedback and intervention.	Moderate<br><br>(Limited details on coding schemes and analysis)
	Chen et al (2014)	General Disasters<br><br>Qualitative-Content Analysis/ Governmental Weibo [Twitter]	When using Weibo in conducting ERC, the Government should increase interaction between the Weibo and its followers to improve mutual communication, initiate conversations, close the gap between information needs and supply, and facilitate its use as a public forum.<br><br>To allow for immediate response and ensure information accuracy, it is crucial to develop a database and to enhance capacity to monitor public opinions on Weibo and to integrate its management into routine government function.<br><br>It is also crucial to offer professional training and support for communicators in charge of Weibo, and to establish an emergency response coordination mechanism among government agencies. This may be followed up with performance evaluation of Weibo, assessing its effectiveness during normal times and in the preparation for, response to and recovery from public health emergencies	Moderate<br><br>(Limited details on coding schemes and analysis)
	Liu et al (2013)	General Disasters<br><br>Qualitative-Content Analysis/ Governmental Weibo [Twitter]	In control of online rumors spreading on Weibo, government agencies need to (1) have robust regulations on rumor control; (2) establish an effective monitoring system; (3) proactively monitor rumors, identify sources of rumors, and intervene when necessary; (4) communicate early and timely updates, respond to rumors immediately with correct information to build public trust in government	Moderate<br><br>(Limited details on coding schemes and analysis)
	The People's Public Opinion Monitoring Office and Weibo Data Center (2012-2016)	General Disasters<br><br>Quantitative- Usage data and Qualitative-Content	Government Weibo has been a part of standard emergency response mechanism. News release and servicing have become the developing trend of government micro blogs. (1) More government officials should utilize Weibo as a service and communication platform. (2) Communication and presentation	Moderate<br><br>(Limited details on coding schemes and

Thematic area of practice	Citation	Type of Emergency Study Methods	Summary findings	CERQual assessment of confidence in the evidence
**Utilization of government controlled Chinese Twitter "Weibo" for ERC** *(continued)*		Analysis/ Governmental Weibo [Twitter]	skills are key to successful information dissemination and these needs to follow the current trend. (3) Adopt a "Government Service Weibo" + "Collective Management" + "Performance evaluation" three pronged mechanism to optimize the efficiency and effectiveness of government services. (4) Diversify government services offered through Weibo and build the "e-service" database to offer one-stop shop. (5) Expand and improve local government's Weibo use to improve reach and impact. (6) Build a positive environment, and monitor/guide public opinions.	analysis)
	Zeng et al (2015)	General Disasters<br><br>Mixed-Methods/ Governmental Weibo [Twitter]	Aided by the new media, government's ERC efforts should (1) evolve away from the traditional top-down approach to two-way exchange of ideas/opinions, and (2) proactively monitor/seek public feedback and make immediate and timely adjustments to ERC strategies	Moderate<br><br>(Limited details on coding schemes and analysis)
	Zhang XE et al (2015)	General Disasters<br><br>Mixed-Methods/ Governmental Weibo [Twitter]	Weibo enabled timely monitoring of other outbreak-relevant information, provided access to additional crowd-sourced epidemiological information and was leveraged by the local government as an interactive platform for risk communication and monitoring public sentiment on the policy response. There is potential for social networking sites to be used by public health agencies to enhance traditional communicable disease surveillance systems for the global surveillance of overseas public health threats. Social networking sites also can be used by governments for calibration of response policies and measures and for ERC. In summary, 1) it may be beneficial for public health agencies to recruit and maintain a workforce of epidemiologists who are multilingual for international disease surveillance in a foreign language. 2) there is significant potential for social media surveillance to be incorporated into mainstream disease surveillance and response systems	Moderate<br><br>(Limited details on coding schemes and analysis)
	Zhou et al (2015)	General Disasters<br><br>Qualitative-Content Analysis/ Governmental Weibo [Twitter]	The Chinese Government Weibo has been an integral part of the response during recent national and regional emergencies. It should play a leadership role in the ERC efforts and ensure: 1) Timely dissemination of the risk information improves public participation, interaction and influence that could guidance public opinion, 2) learn from best practices to improve public impact and ensure openness, transparency, and accountability, and 3) be people-oriented: understand and address the public's information needs, and fulfill social responsibility and guide public opinions	Moderate<br><br>(Limited details on coding schemes and analysis)

Thematic area of practice	Citation	Type of Emergency Study Methods	Summary findings	CERQual assessment of confidence in the evidence
**Collaboration between the US and Chinese CDCs to build ERC capacity**	Hao et al (2009)	General Disasters<br><br>Quantitative Survey	To integrate ERC into national and international public health emergency preparedness planning and response activities, public health agencies such as CDC should have its own spokesperson and media relations trainings and protocols need to be developed and provided to spokesperson prior to disasters.	Moderate<br><br>(Limited details on survey items, process, and analysis)
	Ma et al (2010)	Infectious disease outbreak<br><br>Quantitative Survey	During a disease outbreak, Government must 1) update the public on disease characteristics and prevention methods, 2) identify access to government guidelines, and 3) provide channels for further risk information and situational updates.	Moderate<br><br>(Limited details on survey items, process, and analysis)
	Shao et al (2014)	General Disasters<br><br>Mixed-Methods	To integrate ERC into national and international public health emergency preparedness planning and response activities, China MoH has developed an online training tool for ERC personnel across the country. This effort is to overcome the financial and geographic barriers and disparities, yet a few key elements need to be noted: 1. The online training tool should tailor to users' online habits and combine search engine functionality, information exchange platform, experts' forum in one site. 2. To encourage participation, the tool should allow for interaction between trainers and trainees and offer education credits. 3. Build case bank. 4. Have a live chat option for help and for immediate guidance from ERC experts. 5. It should be complemented with in-person trainings, which offer interaction and immediate Q&A feedback. 6. Algorithms should be used to monitor and analyze usage data in real-time, thereby optimizing user experience and quality of training.	Moderate<br><br>(Limited details on data collection process, and analysis)
	Song et al (2016)	General Disasters<br><br>Quantitative Survey	To integrate ERC into national and international public health emergency preparedness planning and response activities and enhance national and local ERC capacity, it will be important to 1. Create a media monitoring position with designated roles and responsibilities at each CDC office; 2. Provide funding support and equipment; 3. Adopt a nation-wide media monitoring system, managed by central CDC; 4. Enhance media monitoring training; 5. the superior office can direct support to local CDC offices that do not have the capacity to conduct their own media monitoring activities	Moderate<br><br>(Limited details on survey items, process, and analysis)
	Xie et al (2011)	General Disasters	To enhance the national and local ERC capacity, it is important to clarify roles and responsibilities of responding agencies. In China, generally all agreed that the Chinese MoH, especially its emergency management offices, should take	Moderate<br><br>(Limited details on

Thematic area of practice	Citation	Type of Emergency Study Methods	Summary findings	CERQual assessment of confidence in the evidence
**Collaboration between the US and Chinese CDCs to build ERC capacity** *(continued)*		Qualitative	the responsibility of coordination and leading ERC preparedness and response.<br><br>To enhance ERC capacity building for public health workers at all levels of governments, appropriate training, exercises, resources and tools including pre-designed communication supplies (such as posters, brochures, warning signs, etc.), and checklists must be provided.<br><br>Responding agencies should designate a point of contact; identify communication channels and information sharing mechanism. There is also a need to improve public opinions monitoring capacity and promote better cooperation across agencies	data collection process, and analysis)
	Zhang et al (2011)	General Disasters<br><br>Qualitative	It is crucial to develop ERC guidelines for government spokespersons, medical and public health workers, and relevant administrative agency personnel at the local level and integrate them into national and local emergency preparedness plans.<br><br>It is also crucial to develop and offer capacity building training to government spokespersons, medical and public health workers, and relevant administrative agency personnel at the local level and have them participate in scenario-based tabletop and functional exercises Population-based ERC education is also important.	Moderate<br><br>(Limited details on data collection process, and analysis)
**Integration of national health hotline into the emergency preparedness & response system**	Jiang et al (2012)	General Disasters<br><br>Quantitative/ Official public health hotline [12320]	The official public health hotline could be a trusted information source that acts as a direct two-way communication channel between the government and the public during a crisis. Communication by the public. It offered health consultation directly to the public, acted as an important emergency risk communication agent and had taken on the role to gather public reactions data. It has been highly valued by the China MoH and helped shape the ERC strategies.	Moderate<br><br>(Limited details on data collection process, and analysis)
	Wang et al (2010)	General Disasters<br><br>Quantitative	To develop plans and protocols to further integrate the official public health hotline into ERC infrastructure across the country to monitor public opinions and enhance two-way communicate with the public during a health crisis.	Moderate(Limited details on data collection process, and analysis)
