# Supplementary material for: Integrating emergency risk communication (ERC) into the public health system response: Systematic review of literature to aid formulation of the 2017 WHO Guideline for ERC policy and practice
Source: PLoS One. 2018 Oct 31;13(10):e0205555. doi: 10.1371/journal.pone.0205555 (PMC6209198; doi:10.1371/journal.pone.0205555)
Supplement: S3 Table — (PDF) [file pone.0205555.s003.pdf]

S3 Table: Individual study findings within methodological streams and evaluation of confidence – Questions 2 & 3 (combined)

Thematic area of practice	Citation	Type of Emergency<br>Study Methods<br>Country/Geographic Context	Summary findings	CERQual assessment of confidence in the evidence
**Networks to enhance information sharing**	Gresham L., Ramlawi A, Briski J, Richardson M, Taylor T. Trust across borders: responding to 2009 H1N1 influenza in the Middle East. *Biosecurity and Bioterrorism: Biodefense Strategy, Practice, and Science.* Volume 7, Number 4, 2009.	Pandemic Influenza<br><br>Case study<br><br>Middle East	Regional disease surveillance networks may provide a useful mechanism for information sharing as described by Gresham et al (2009) the Middle East Consortium on Infectious Disease Surveillance (MECIDS), a regional disease surveillance network of public health experts and ministry of health officials from Israel, the Palestinian Authority, and Jordan. MECIDS unites public health officials of differing Middle Eastern nationalities and contributes to regional health and stability by engaging in regular cross-border information exchange, conducting regular executive board meetings, performing laboratory and risk communications training, and implementing innovative communication technology. Initially focused on food- and waterborne diseases, the partnership developed a network of laboratories, protocols for specimen collection and diagnosis of diarrheal diseases, and data sharing and notification capabilities, so as to analyze and share information on disease threats. During H1N1, the MECIDS partners agreed to prompt and coordinated border and airport screening, laboratory testing, information exchange, and common communication strategies. This coordination can be largely contributed to the existence of both trust and well-exercised national and regional pandemic preparedness plans, which were initially established within this network.	Moderate<br><br>(Minor concerns on adequacy of data)
	Moore M, Dausey D. Response to the 2009-H1N1 influenza pandemic in the Mekong Basin: surveys of country health leaders. *BMC Research Notes.* 2011, 4:361.	Pandemic Influenza<br><br>Survey<br><br>Asia	Regional disease surveillance networks may provide a useful mechanism for information sharing. As described by Moore and Dausey (2009) the Mekong Basin Disease Surveillance (MBDS) is a cooperating network, comprised of six countries - Cambodia, China (originally just Yunnan province and, since 2008, Guangxi Province as well), Lao People's Democratic Republic, Myanmar, Thailand and Vietnam - which formally organized themselves in 2001 to collaborate on sub-regional infectious disease surveillance and control. During the H1N1 pandemic, MBDS health leaders perceived their pandemic responses to be effective in areas that, prior to the creation of the network, were considered problematic. Most felt that MBDS cooperation helped drive such efforts, and thus added value. Surveillance capacity within countries and surveillance information sharing across countries, a longstanding MBDS focus areas, were cited as strengths. Participants noted the ability of their country level surveillance systems to exchange information efficiently within the country and the importance of MBDS for enabling timely coordinated regional response to detect disease at cross border sites and prevent the spread of the virus across countries.	Moderate<br><br>(Minor concerns on methodology and adequacy of data)

Thematic area of practice	Citation	Type of Emergency<br>Study Methods<br>Country/Geographic Context	Summary findings	CERQual assessment of confidence in the evidence
**Task Forces and Committees (TFC) to facilitate information sharing**	Chess C., Clarke L. Facilitation of Risk Communication During the Anthrax Attacks of 2001: The Organizational Backstory. Weapons of Mass Destruction. *American Journal of Public Health.* 2007; 97:1578-1583.	Anthrax<br><br>Interviews<br><br>USA	Task forces can facilitate interaction between professionals belonging to different organizations as described by Chess et al (2007), in this case a bioterrorism task force was established in a county in New Jersey before the anthrax attacks, and how it served as a mechanism to create a network of people who trusted each other during the Anthrax incidents. Successful risk communication in a health crisis is often contingent upon effective communication among agencies, and organizational and professional networks were essential to risk communications efforts. Frequent interaction among a diverse group of professionals and, in particular, between law enforcement and public health officials, facilitated the creation of consistent messages. Where law enforcement and health agencies had preexisting, cooperative relationships, there was often a commitment to compromise, accommodating differing goals and methods.	Low<br><br>(Significant concerns on methodology and adequacy of data)
	Clarke C. and Chess C. False Alarms, Real Challenges – One University's Communication Response to the 2001 Anthrax Crisis. *Biosecurity and Bioterrorism: Biodefense Strategy, Practice and Science.* Volume 4, Number 1, 2006.	Anthrax<br><br>Interviews<br><br>USA	The creation of an Emergency Operations Committee (EOC) can facilitate the sharing of information. Clarke et al (2006) describes how, during the 2001 Anthrax attacks, a university in the US organized an Emergency Operations Committee (EOC) designed to bring together groups such as Police, EHS, Occupational Health, and other departments, to share information, pool resources, and coordinate communication with the university community. Collaboration involved the pooling of resources, sharing of information, and integration of communication infrastructure to form a more organized approach to risk communications.	Moderate<br><br>(Minor concerns on methodology and adequacy of data)
**Elements of functionality of networks and task-forces**	Nowell B, Steelman T. Communication under Fire: The Role of Embeddedness in the Emergence and Efficacy of Disaster Response Communication Networks. Journal of Public Administration Research and Theory. 2015;25(3):929-52.	Wildfires<br><br>Case Study<br><br>USA	Nowell et al. (2015*)* underlines the importance of interpersonal relationships, through the use of dyadic network analysis, in a US based study of institutional and relational embeddedness in relation to the response to wildfires. Responders are significantly more likely to interact more frequently with people with whom they have existing relationships (familiarity/relational embeddedness), even after controlling for similarities in role function and stakeholder group. This suggests that efforts by emergency managers and other key stakeholders to establish working relationships among responders before an incident will increase the likelihood that those responders will stay in communication with one another during an actual incident.	Low<br><br>(Significant concerns on methodology and adequacy of data)
	Schraagen JM, Veld MHit, De Koning L. Information Sharing During Crisis Management in	Terrorism<br><br>Controlled laboratory	In simulated environments, network teams work faster and arrive at more correct decisions than hierarchical teams as described by Schraagen et al (2010). Network structures allow teams to exchange information quickly, monitor each other's	Moderate<br><br>(Minor concerns on

Thematic area of practice	Citation	Type of Emergency Study Methods Country/Geographic Context	Summary findings	CERQual assessment of confidence in the evidence
**Elements of functionality of networks and task-forces** *(continued)*	Hierarchical vs. Network Teams. Journal of Contingencies and Crisis Management. 2010;18(2):117-27.	experiment comparing team structures<br><br>Netherlands	performance, and build mutual trust. Network teams were found to perform faster than hierarchical teams, while maintaining the same level of accuracy in relatively simple environments. In relatively complex environments, network teams arrive at correct decisions more frequently than hierarchical teams.	methodology)
	Bharosa N, Lee J, Janssen M. Challenges and obstacles in sharing and coordinating information during multi-agency disaster response: Propositions from field exercises. Information Systems Frontiers. 2010;12(1):49-65.	General Disasters<br><br>Case study<br><br>Netherlands	The use of information manager can facilitate cross-agencies information sharing as suggested by Bharosa et al (2010) in a case study on a multi-agency disaster management exercise in the Port of Rotterdam (POR), the Netherlands in order to collect data on disaster management and information sharing. Unlike its American counterpart, the Dutch Emergency Response Center (ERC), acts as an information center for just one relief agency. However, the presence of a central hub can act as a single point of failure, as it possesses limited knowledge about ongoing emergency response activities, resulting in uncontrolled information filtering/dissemination and a lack of direct information exchange between some information users and information sources. Managing some of the information flows between the decision-making units (COPI, ROT) and the ERC is usually the job of an Information Manager (IM), a role usually filled by a police officer, who is responsible for providing specific information services to decision-making units.	Moderate<br><br>(Minor concerns on methodology and adequacy of data)
	Howard AF, Bush HM, Shapiro RM, 2nd, Dearinger A. Characteristics of Kentucky local health departments that influence public health communication during times of crisis: information dissemination associated with H1N1 novel influenza. J Public Health Manag Pract. 2012;18(2):169-74. Epub 2012/01/31.	2009 H1N1 outbreak<br><br>Survey<br><br>USA	Public Information Officers (PIOs) can facilitate effective information dissemination. Howard et al (2012) describe the importance of having a Public Information Officer (PIO) at the local public health department level to facilitate effective information dissemination to health care professionals. In this analysis, the absence of a PIO was associated with decreased communication with those agencies external to the LHD.	Low<br><br>(Significant concerns on adequacy of data)
	Holmes BJ, Henrich N, Hancock S, Lestou V. Communicating	Emerging infectious	Early engagement of media can enhance information dissemination. Holmes et al (2009) presents the findings of interviews conducted with communications directors	Moderate

Thematic area of practice	Citation	Type of Emergency<br>Study Methods<br>Country/Geographic Context	Summary findings	CERQual assessment of confidence in the evidence
**Elements of functionality of networks and task-forces**<br><br>*(continued)*	with the public during health crises: Experts' experiences and opinions. Journal of Risk Research. 2009;12(6):793-807.	diseases<br><br>Interviews<br><br>Canada	and public health officials in British Columbia on components to effective communication. They describe the importance of engaging media representatives immediately in discussions about potential emerging infectious disease outbreaks, including the role media should play and how the public health community can help them fulfill that role.	(Minor concerns on methodology and adequacy of data)
**Use of information systems to enhance ERC**	Celik S., Crbacioglu S. Role of information in collective action in dynamic disaster environments. *Disasters* 2010, 34(1):137-154.	Earthquake<br><br>Interviews and daily analysis of reports<br><br>Turkey	The use of information systems impacts timeliness in the response to earthquakes. Celik et al (2010) describe the response to two Earthquakes in Turkey. Turkish disaster experts claimed that the presence of information systems provided timely and accurate information during the Duzce earthquake operations, whereas a lack of information systems created problems in obtaining timely and accurate information for coordinated action during the Marmara earthquake operations. Consequently, the Turkish disaster management system was better prepared three months later, when the Duzce earthquake hit, and performed better in response operations with reduced difficulty in accessing and exchanging information in an accurate and timely manner, so as to facilitate coordination and search-and-rescue activities. Improvements to the information infrastructure, investments in information technology, such as a more effective use of wireless communications, and changes in behavior through individual and organizational learning increased the capacity for information searching, acquisition, and exchange. A better understanding of the importance of disaster-relevant information, along with experience of the Marmara earthquake, helped facilitate more effective use of available communication means for information sharing in response to the Duzce earthquake and assisted in achieving a faster, more coordinated response operation.	Low<br><br>(Significant concerns on adequacy of data)
	Seyedin SH., Jamil HR. Health Information and Communication System for Emergency Management in a Developing Country, Iran. *J Med Syst*. 2011; 35:591-597.	All hazards<br><br>Survey and interviews<br><br>Iran	Information systems need to be tailored to the needs of users which may differentiate across roles and responsibilities in disaster planning and response. In this article, recommendations are provided for the creation of an information system based on the experience of interviewees. Interviewees reported the need for the creation of five different information databases for emergency management (EM). These databases are: demographics; disasters; senior and line managers; hospitals and therapeutic facilities, laboratories and emergency posts, and health facilities, such as health centers. The authors noted that managers at local level had a different understanding of an EM information system compared to managers at the national level. While participants at	Low<br><br>(Significant concerns on adequacy of data)

Thematic area of practice	Citation	Type of Emergency<br>Study Methods<br>Country/Geographic Context	Summary findings	CERQual assessment of confidence in the evidence
**Use of information systems to enhance ERC**<br><br>*(continued)*			the regional and national levels mainly saw an EM database from a preparedness perspective, the majority of managers at the local level focused on the response phase and requirements that an effective information system needs to meet at this phase, such as logging, informing and information broadcasting in response to disasters. An important issue in the EM information system is analysis of available data in order to produce sensible information for decision making and management purposes.<br><br>Using a website to broadcast the information of casualties, such as their photos, place of admission, and regularly updating such websites psychologically relieves and reduces the stress of families and relatives, as well as reducing unnecessary transportation and pressure on communication infrastructures.	
	Ipe M., Raghu TS., Vinze A. Information intermediaries for emergency preparedness and response: A case study from public health. *Inf Syst Front.*2010; 12:67-79.	All hazards<br><br>Interviews<br><br>USA	The creation of information systems for surveillance and intelligence may facilitate information sharing. Ipe et al (2010) document a new public health surveillance system, the Medical Electronic Disease Surveillance and Intelligence System (MEDSIS) in Arizona (USA). MEDSIS is a web-based application designed to enable public health agencies to detect and respond to bioterrorism events, outbreaks of infectious diseases, and other public health emergencies, and places the state agency in the role of an information intermediary.	Moderate<br><br>(Minor concerns on methodology)
	Bharosa, N. A Case Study of Information Flows in Multi-Agency Emergency Response Exercises. 2009 January. The proceedings of the 10th International Digital Government Research Conference.	Flooding<br><br>Case Study - interviews, observations and document/archival analysis<br><br>Netherlands/Germany	Information systems used by bordering countries facilitate information sharing. Bharosa et al. describe how, in order to improve collaboration and information sharing between relief agencies at the Dutch-German border, the Province of Gelderland (Netherlands) and the Province of Nordrhein-Westfalen (Germany) have joined forces and organized annual cross-border multi-agency exercises on flood management since 2004. The collaboration program is labeled as VIKING, an acronym for improving information sharing in times of flood management at the Dutch-German border. During the exercise, both the Dutch agencies and the German agencies use their own proprietary information and communication systems, except for one common system: a flood management system called FLIWAS, standing for Flood Information and Warning System. FLIWAS is a high-level water control application, built as a Dutch-German cooperation, and is funded by the EU. The purpose of this application is to optimize the exchange of information during high water situations within and between water management and calamity management organizations. This application is built using generic components (e.g. communication with databases, internal and external communication, import provider, plan module and web mapping). FLIWAS plays a	Moderate<br><br>(Minor concerns on methodology and adequacy of data)

Thematic area of practice	Citation	Type of Emergency<br>Study Methods<br>Country/Geographic Context	Summary findings	CERQual assessment of confidence in the evidence
**Use of information systems to enhance ERC**<br><br>*(continued)*			central role in information sharing across the Dutch-German border.	
	Leonard GS., Stewart C., Wilson TM., Procter JN., Scott NJ., Keys HJ., Jolly GE., Wardman JB., Cronin SJ., McBride SK. Integrating multidisciplinary science, modeling and impact data into evolving, syn-event volcanic hazard mapping and communication: A case study from 2012 Tongariro eruption crisis, New Zealand. *Journal of Volcanology and Geothermal Research.* 2014. (286): 208-232.	Volcano eruption<br><br>Case study<br><br>New Zealand	Common information sources across disciplines acoompained by pre-existing relationships facilutate the flow of information during an emergency. Leonard et al describe response to the 2012 Togariro eruption in New Zealand and describe the main science communication mechanims used, including the communication framework and the inter-agency relationships in which the crisis hazard maps were being developed. The authors analyzed communications amongst scientists (through the use of a wiki technical clearinghouse), communications with emergency managers, communicatiosn with human health and agricultural agencies, communications with critical infrastructure agencies and communications with media and the public. This crisis demonstrates the value of pre-existing relationships, having ready-to-go information resources, and the substantial time and resources required for communications.	Moderate<br><br>(Minor concerns on methodology)
	Kapucu N. Interagency Communication Networks During Emergencies Boundary Spanners in Multiagency Coordination. *American Review of Public Administration*. 2006. 36 (2):207-225.	Terrorism<br><br>Content Analysis & Interviews<br><br>USA	Information systems shared across agencies enhance response capabilities. Kapucu et al (2006) describe barriers to effective decision making in response to the WTC terrorist attacks in New York City, and the importance of a multi-organizational communication and coordination response from emergency management. The New York Police Department (NYPD)'s 9-1-1 operators and the Fire Department New York (FDNY) dispatch were not adequately integrated into the emergency response. Inadequate communication also hindered the Port Authority's response. The lack of both communication and an integrated system hindered agencies' communication and decision making. In future disasters, it is critical to analyze how responding agencies, victims, and the public will get information and help.	Moderate<br><br>(Minor concerns on methodology and adequacy of data)

Thematic area of practice	Citation	Type of Emergency<br>Study Methods<br>Country/Geographic Context	Summary findings	CERQual assessment of confidence in the evidence
**Use of information systems to enhance ERC**<br><br>*(continued)*	Militello LG, Patterson ES, Bowman L, Wears R. Information flow during crisis management: challenges to coordination in the emergency operations center. *Cognition, Technology & Work*. 2007;9(1):25-31.	All hazards<br><br>Case study<br><br>USA	Militello et al. discusses the challenges to coordination in the emergency operations centers (EOC). Through two county-level exercises, it was observed that there exists an asymmetry with regards to experience and knowledge of responders across agencies, which in turn creates coordination challenge as issues of trust and cohesion become increasingly important during high-intensity disasters. They suggested that low-cost paper-based tools like notebooks, whiteboards, and telephone books would be extremely useful tools of communication in such EOCs.	Moderate<br><br>(Minor concerns on methodology)
	Chang, 2013.<br>台湾重大灾害灾情查通报系统运作之经验分析<br>[Operations of the Emergency Information Management System in Taiwan].<br>复旦公共行政评论 [Fudan Public Administration Review]. 2013 (2), P. 147-168.	All Hazards/Typhoon<br><br>Survey<br><br>Taiwan	To improve the disaster notification system in Taiwan, it is crucial<br><br>- to diversify the use of communication channels and devices<br><br>- to seek and designate local governmental resources to fund the use of communication devices in the less developed areas in order to address the equality gap among regions<br><br>- it would be important to establish partnership between government agencies and NGOs and integrate the disaster information management system (such as Tsu Chi's DRM and Red Cross' DMIS) to gather public opinions, coordinate and share information<br><br>In Taiwan, the disaster situational information gathering and dissemination is performed by the Fire Department, the Police Department, and the Department of Civil Affairs, as well as being supplemented by an emergency management information system. These official systems carry out cross-institution information sharing. To improve the disaster notification system in Taiwan, it is crucial to identify the agency that has a better coverage, in this case, the police system, and use it to direct crisis information sharing<br><br>Taiwan has developed and implemented a new "Emergency Management Information System"(EMIS) for disaster information sharing, management, and coordination across agencies. Stakeholders suggested to improve EMIS' capacity to be able to monitor and integrate disaster information available on social media, offer timely update on landslides, road interruption, casualty and share information on volunteers, relief assistance and resource available	Moderate<br><br>(Limited details on survey items and process)
	Thiago Reginaldo T. Pacheco DC, Baldessar MJ, Benciveni	Natural disaster	The use of information and communication technologies in the public sector is perceived by the population as a valuable means for offering high quality services,	Moderate

Thematic area of practice	Citation	Type of Emergency<br>Study Methods<br>Country/Geographic Context	Summary findings	CERQual assessment of confidence in the evidence
**Use of information systems to enhance ERC**<br><br>*(continued)*	Franzoni AM, 2013. Redução de riscos de desastres na prática: a participação da Defesa Civil e do município em plataformas digitais de redes sociais Democracia digital e governo eletronico, Capa > v. 2, n. 9 (2013)	Case study<br><br>Brazil	reliable information, and for gaining insight into mechanisms of governance.<br><br>Thiago et al. (2013) describe the use of social networks, like Twitter and Facebook (FB), by the Civil Defense (CD) of New Freiburg, Rio de Janeiro, Brazil, as a communication channel for alerting and preparing the population to face a natural disaster. This study aimed to explore the possible benefits of expanding digital platforms, including web pages, social networks, mobile phone SMS, research engines, video games and digital TV programs, towards a larger community audience, in order to spread messages about the correct behaviors in facing different phases of disasters. Through the analysis of Twitter and Facebook use by CD to communicate with affected population during a specific event, like the flood occurred in 2011 in New Freiburg.<br><br>This study examined the period from 2011-13, so as to track digital activity since the creation of specific CD pages in social networks. Content analysis of messages provided positive feedback on enrollment and mobilization of authorities after the event.<br><br>Following the flood, during the reconstructive phase, the CD and municipality of New Freiburg launched and promoted several public events through social networks, in order to gather, inform, prepare and reassure both the population already affected by the flood and those still possibly at risk. The authors conclude that in a country like Brazil, where network coverage is broad and accessible, it is worth considering the potential for social networks to act as a new institutional channel of communication for health related topics.	(Minor concerns on methodology)
**Local stakeholders' engagement**	Ardalan A., Naieni KH., Kabir MJ., Zanganeh AM., Keshtkar AA., Honavar MR., Khodaie H.,Osooli M. Evaluation of Golestan Province's Early Warning System for flash floods, Iran, 2006-7. *Int J Biometerol* 2009. 53:247-254.	Flooding<br><br>Interviews<br><br>Iran	In this study, the authors use the results of interviews, conducted with experts and citizens, to evaluate the response to flash flood early warning systems in Iran. The authors propose the creation of a "Village Disaster Taskforce" (VDT), in which individual villages act as operational units in the early warning mechanism. This model would overcome the limitations of the current Early Warning System by facilitating the warning dissemination and communication at the grassroots level. It would not replace a high-tech early warning system, but would strengthen the existing system at the local level.	Moderate<br><br>(Minor concerns on adequacy of data)
	Cole JM., Murphy BL. Rural hazard risk communication and public education: Strategic	All hazards<br><br>Interviews	In this study, the authors note that the present literature supports the belief that disaster and emergency management (DEM) public education should be framed for specific target audiences. To accomplish this goal with limited resources, the authors	Moderate<br><br>(Minor concerns on

Thematic area of practice	Citation	Type of Emergency Study Methods Country/Geographic Context	Summary findings	CERQual assessment of confidence in the evidence
**Local stakeholders' engagement** *(continued)*	and tactical best practices. *International Journal of Disaster Risk Reduction*. 2014 (10):292-304	Canada	propose small rural municipalities could consider creating partnerships with neighboring small communities, or use regional organizations, to complete these tasks.<br><br>In order to deal with the problems associated with role sharing and the lack of knowledge regarding local risk perceptions and appropriate disaster risk reduction (DRR) activities, Community Emergency Management Coordinators should consider tapping into existing social networks and identifying community leaders and local organizations who can assist with the development and delivery of DEM public education programs.	adequacy of data)
	Shepherd J., Vuuren Kv., The Brisbane flood: CALD gatekeepers' risk communication role. *Disaster Prevention and Management*. 2014;23(4):469-483.	Flooding<br><br>Australia<br><br>Interviews/case study	Informational material that addresses the need of culturally and linguistically diverse populations should be included in centralized information sources. This study discusses how, during the 2011 Brisbane flood in Australia, some Culturally And Linguistically Diverse (CALD) "gatekeepers" working at the community level encountered problems accessing emergency risk information that matched their communities' needs, despite having access to multiple sources of information. There is a need for a well-promoted and central information source that can be immediately accessed to ensure that emergency risk information can be more effectively communicated to CALD communities.	Moderate<br><br>(Minor concerns on adequacy of data)
	Gultom DI., Joyce Z. Crisis communication capacity for disaster resilience: Community participation of information providing and verifying in Indonesian volcanic eruption. Australian and New Zealand Third Sector Research. November 2014<br><br>Permanent URL:<br><br>http://apo.org.au/node/53138	Volcanic eruptions<br><br>Interviews<br><br>Indonesia	Empowerement of local communities in ERC may be an effective strategy to enhance ERC. This study describes how local communities can empower themselves through participation in providing, sharing, and verifying the information within their social networks. The Merapi Circle Information Networks (Jalin Merapi or JM) is an example of community-based ERC sharing and coordination. JM has developed a collaborative system, with community radio stations and local communities as reliable information sources and direct verifiers, and also utilizes a community's social media capacity. The system is based on community's charcateristics, such as local culture, levels of communication technology adaptation, media pereferences, daily communication behaviors and level of trust among community's members.	Moderate<br><br>(Minor concerns on adequacy of data)
	Mulyasari F., Shaw R. Role of women as risk communicators	All hazard	Women can play an important role in ERC> This study focuses on the risk communications processes in Bandung Indonesia, in which women act as risk	Low

Thematic area of practice	Citation	Type of Emergency<br>Study Methods<br>Country/Geographic Context	Summary findings	CERQual assessment of confidence in the evidence
**Local stakeholders' engagement**<br><br>*(continued)*	to enhance disaster resilience of Bandung, Indonesia. *Nat Hazards.* 2013 69:2137-2160	Survey<br><br>Indonesia	communicators. The results of this study show that most of the Women's Welfare Association (WWA) leaders at the ward level perceive that advocating and cooperating with the ward and sub-district government in establishing early warning systems is necessary prior to a disaster taking place. Due to the unique organizational character of WWAs, where the leaders of women at the city, sub-district, and ward levels are the spouses of the mayor, the sub-district leaders, and the ward leaders respectively, the women's leaders in Bandung have formed an effective working partnership with the local government.	(Significant concerns on methodology and adequacy of data)
	Lei, 2015.<br>高速公路突发事件组织间应急信息沟通实证研究<br>[Empirical Study on the Inter-organizational Communication in the Case of Expressway Emergency]. Management Review. 2015, Vol. 27 Issue (9): 213-220. DOI:10.14120/j.cnki.cn11-5057/f.2015.09.019	Major Accident<br><br>Semi-structured interviews and content analysis<br><br>China	To improve the response and management of highway emergencies, Lei (2015) suggested:<br><br>- to involve both governmental agencies and NGOs in the information sharing and coordination mechanism<br><br>- to develop an evidence-based, scenario-based contingency plan, including communication plans involving respective responding agencies and case studies on various types of scenarios, including highway emergencies<br><br>- to develop communication plans that are scenario-based and include details of how agencies not involved in daily highway operations can coordinate and share information with operations agencies.<br><br>- to include communications with lay people, who are unfamiliar with highway operations or emergency response, in capacity building training for responders<br><br>- to build a platform for emergency information sharing and offer technical support for various parties involved<br><br>- to ensure the burden of information processing is manageable, balanced, and within the capacity of agency that handles it<br><br>- to incorporate mandatory time off in personnel timetable to improve sustainability and stability.	Moderate<br><br>(Limited details on interview items, process as well as coding and process for content analysis)
